# A Massively Parallel Pipeline to Clone DNA Variants and Examine Molecular Phenotypes of Human Disease Mutations

**DOI:** 10.1371/journal.pgen.1004819

**Published:** 2014-12-11

**Authors:** Xiaomu Wei, Jishnu Das, Robert Fragoza, Jin Liang, Francisco M. Bastos de Oliveira, Hao Ran Lee, Xiujuan Wang, Matthew Mort, Peter D. Stenson, David N. Cooper, Steven M. Lipkin, Marcus B. Smolka, Haiyuan Yu

**Affiliations:** 1 Department of Medicine, Weill Cornell College of Medicine, New York, New York, United States of America; 2 Weill Institute for Cell and Molecular Biology, Cornell University, Ithaca, New York, United States of America; 3 Department of Biological Statistics and Computational Biology, Cornell University, Ithaca, New York, United States of America; 4 Department of Molecular Biology and Genetics, Cornell University, Ithaca, New York, United States of America; 5 Institute of Medical Genetics, Cardiff University, Heath Park, Cardiff, United Kingdom; Stanford University School of Medicine, United States of America

## Abstract

Understanding the functional relevance of DNA variants is essential for all exome and genome sequencing projects. However, current mutagenesis cloning protocols require Sanger sequencing, and thus are prohibitively costly and labor-intensive. We describe a massively-parallel site-directed mutagenesis approach, “Clone-seq”, leveraging next-generation sequencing to rapidly and cost-effectively generate a large number of mutant alleles. Using Clone-seq, we further develop a comparative interactome-scanning pipeline integrating high-throughput GFP, yeast two-hybrid (Y2H), and mass spectrometry assays to systematically evaluate the functional impact of mutations on protein stability and interactions. We use this pipeline to show that disease mutations on protein-protein interaction interfaces are significantly more likely than those away from interfaces to disrupt corresponding interactions. We also find that mutation pairs with similar molecular phenotypes in terms of both protein stability and interactions are significantly more likely to cause the same disease than those with different molecular phenotypes, validating the *in vivo* biological relevance of our high-throughput GFP and Y2H assays, and indicating that both assays can be used to determine candidate disease mutations in the future. The general scheme of our experimental pipeline can be readily expanded to other types of interactome-mapping methods to comprehensively evaluate the functional relevance of all DNA variants, including those in non-coding regions.

## Author Summary

With rapid advances in sequencing technologies, tens of millions of DNA variants have now been discovered in the human population. However, there are currently no experimental methods available for examining the impact of DNA variants in a high-throughput fashion. As a result, we have no functional data on the vast majority of these variants, which is a major roadblock to generating novel biological insights and developing new disease prevention therapeutic strategies. To address this issue, we have successfully developed the first massively-parallel site-directed mutagenesis approach, Clone-seq, to leverage the power of next-generation sequencing to generate a large number of mutant alleles in a fast and cost-effective manner. In conjunction with Clone-seq, we established a high-throughput comparative interactome-scanning pipeline to experimentally elucidate the effect of variants on protein stability and interactions. Additionally, Clone-seq can be used to generate clones for all DNA variants, including those in non-coding regions.

## Introduction

Owing to rapid advances in next-generation sequencing technologies, tens of thousands of disease-associated mutations [1] and millions of single nucleotide polymorphisms (SNPs) [2], [3] have been identified in the human population. With the large number of ongoing whole-exome and whole-genome sequencing projects [2], [3], hundreds of thousands of new SNPs are now being discovered every month. Hence, there is an urgent need to develop high-throughput methods to sift through this deluge of sequence data and rapidly determine the functional relevance of each variant. Here, we focus on coding variants, firstly because trait- and disease-associated SNPs are significantly over-represented in nonsynonymous sites [4], and secondly because the vast majority of disease-associated mutations identified to date reside within coding regions [1]. We evaluate the functional impact of coding variants by examining their effects on corresponding protein-protein interactions, because most proteins carry out their functions by interacting with other proteins [5].

Recent studies have begun to use large-scale protein interaction networks to understand human diseases and their associated mutations [5], [6]. By integrating structural details with high-quality protein networks, we created a 3D interactome network where the interface for each interaction has been structurally resolved [7]. Using this 3D network, we demonstrated that in-frame disease mutations (missense mutations and in-frame insertions/deletions) are significantly enriched at the interaction interfaces of the corresponding proteins [7]. Our results indicate that alteration of specific interactions is very important for the pathogenesis of many disease genes, highlighting the importance of 3D structural models of protein interactions in understanding the functional relevance of coding variants. However, many important questions still remain unanswered – for example, what fraction of protein-protein interactions is altered by disease mutations to cause the corresponding disorders? Furthermore, do structural details of the interacting proteins, especially the position of the mutation relative to the interaction interface, affect the ability of a given disease mutation to alter a specific interaction?

**Figure 1:**
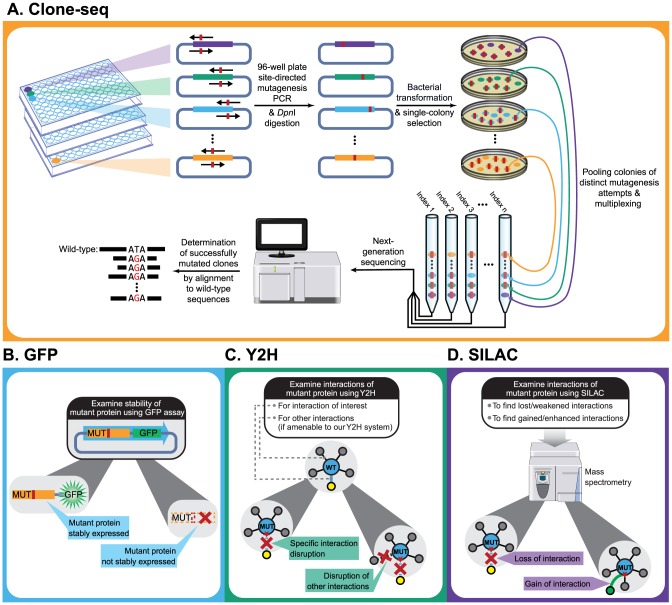
Schematic of our comparative interactome-scanning pipeline. Our pipeline begins with Clone-seq (a), a massively-parallel low-cost site-directed mutagenesis pipeline leveraging next-generation sequencing. This is followed by a high-throughput GFP assay (b) to determine protein stability, and a high-throughput Y2H assay (c), along with SILAC-based mass spectrometry (d) to determine the impact of DNA coding variants on protein interactions.

To address these questions, we decided to focus on proteins with known disease mutations that participate in interactions with available co-crystal structures in the Protein Data Bank (PDB) [8]. To detect the alteration of the interactions by disease mutations, it is necessary to first detect the interactions of the wild-type proteins using an assay of choice. This turns out to be a major bottleneck because all high-throughput interaction-detection assays have very limited sensitivity [9], [10]. Our assay of choice is Y2H because there are over 16,000 human protein interactions detected by our version of Y2H that can serve as the reference interactome for comparison [11], [12], [13], [14], the largest for any assay performed to date (**Figure S1**). In total, there are 217 interactions detected by our version of Y2H with available co-crystal structures; 51 of these also have known missense disease mutations on corresponding proteins in the Human Gene Mutation Database (HGMD) [1] and the corresponding interactions for the wild-type proteins can be detected in our experiments with strong Y2H-positive phenotypes (**Figure S2**; **Materials and Methods**). Here, we focused on missense mutations because they are intrinsically more likely to generate interaction-specific disruptions [6]. We established a high-throughput comparative interactome-scanning pipeline to clone disease mutations and examine their molecular phenotypes (**Fig. 1**). The methodologies established here can be readily applied to any non-synonymous variant in the coding region, including nonsense mutations.

## Results

### Clone-seq: A massively parallel site-directed mutagenesis pipeline using next-generation sequencing

**Figure 2:**
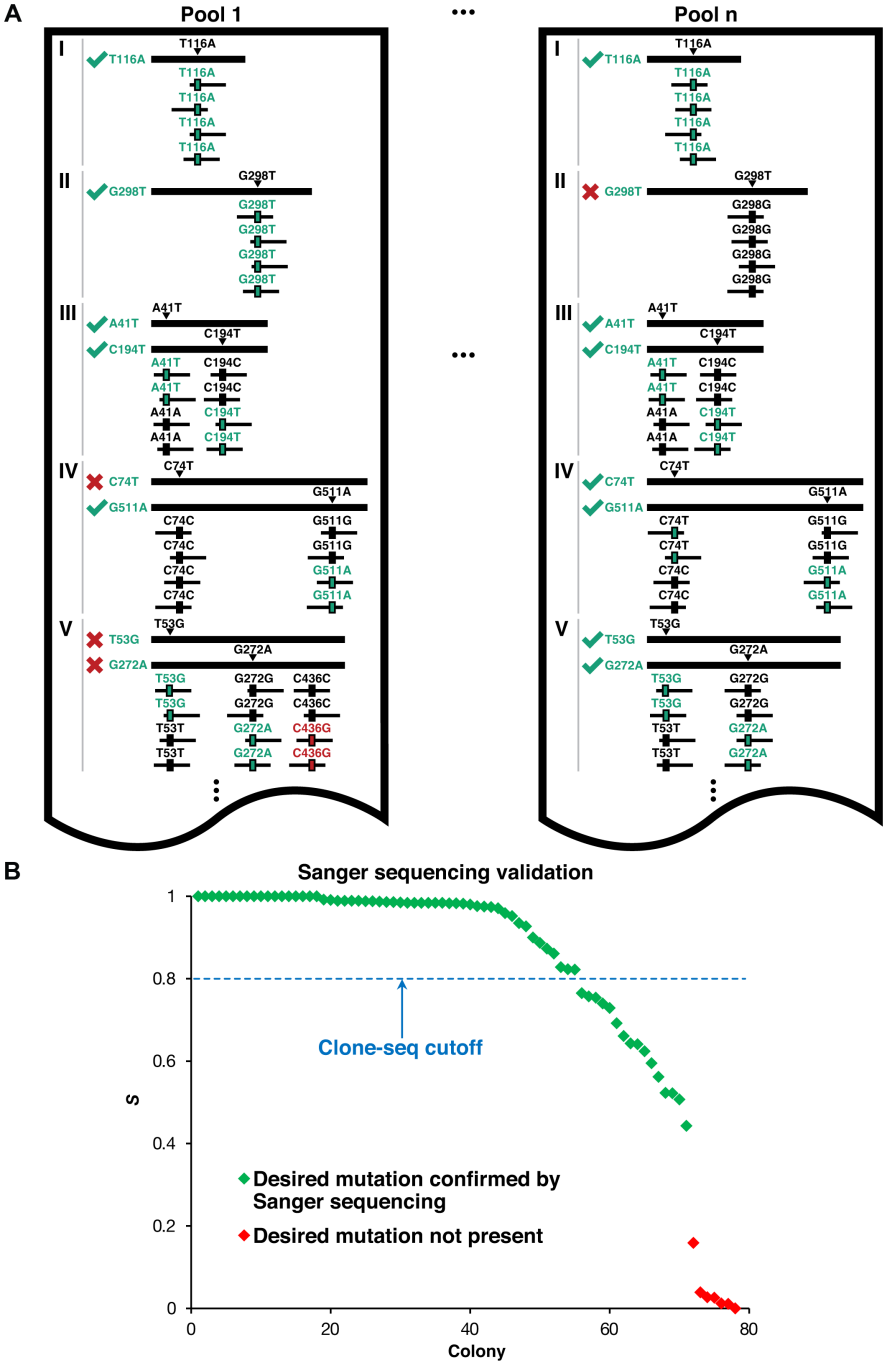
Identifying usable clones from Clone-seq. (a) Schematic illustrating criteria used to determine which of the clones generated by our Clone-seq pipeline are usable for further assays – green ticks indicate usable clones, while red crosses indicate clones that cannot be used. (b) Variation of *S* across different mutagenesis attempts that either contain or do not contain the desired mutation as confirmed by Sanger sequencing.

The first step of our pipeline is a massively parallel approach, termed Clone-seq, designed to leverage the power of next-generation sequencing to generate a large number of mutant alleles using site-directed mutagenesis in a rapid and cost-effective manner. Current protocols for site-directed mutagenesis require picking individual colonies and sequencing each colony using Sanger sequencing to identify the correct clone [15]. This standard approach is both labor-intensive and expensive; therefore, it does not scale up to genome-wide surveys. In Clone-seq, we put one colony of each mutagenesis attempt into one pool (**Fig. 1a**; in other words, each pool contains one and only one colony for each desired mutation) and combine multiple pools through multiplexing for one Illumina sequencing run [16]. Colonies for generating different mutations of the same gene can be put into the same pool, which can be easily distinguished computationally when processing the sequencing results. This is true even for mutations occurring at the same site (**Fig. 2a**, **Text S1**).

**Figure 3:**
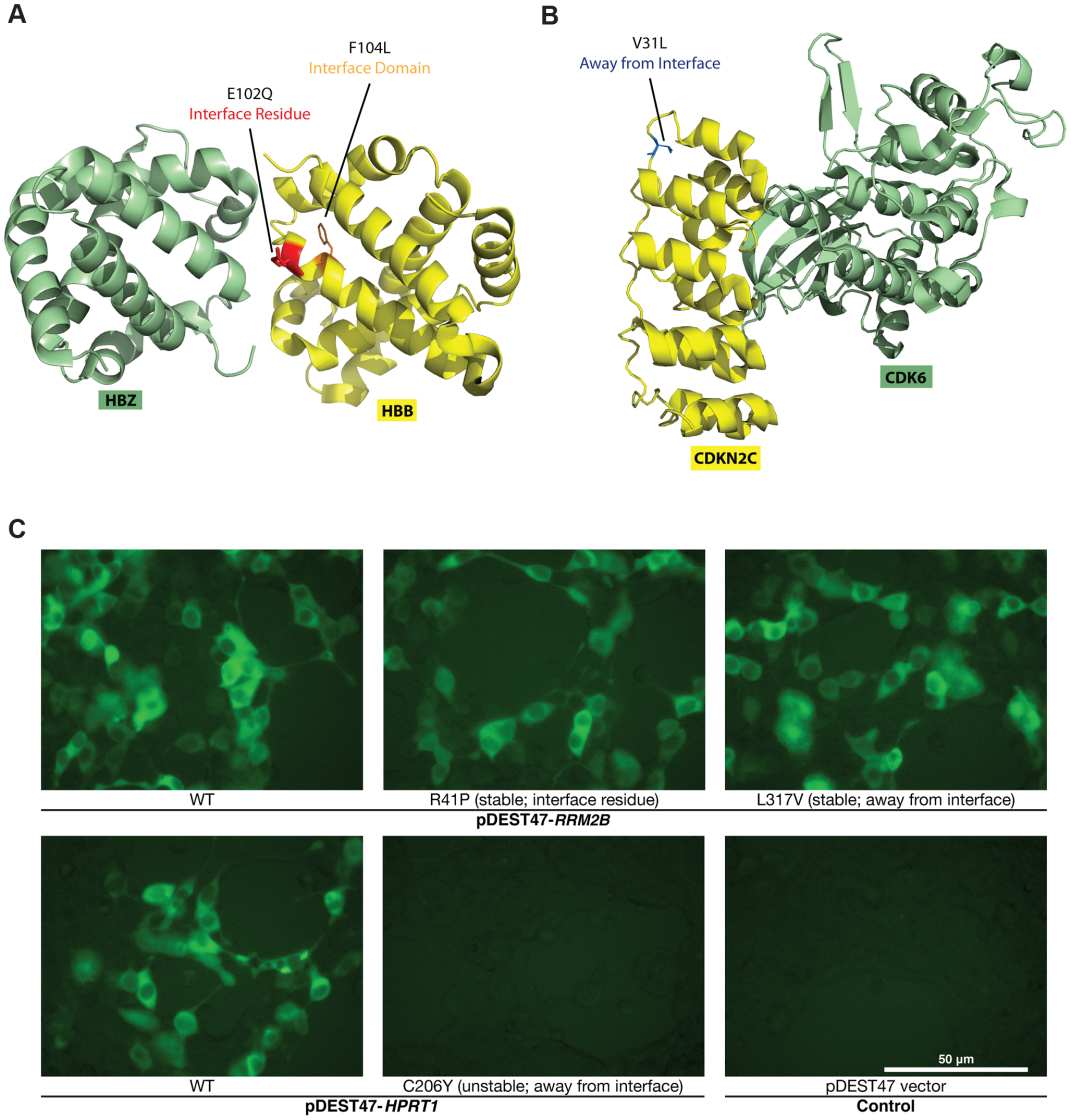
Examples of disease mutations in different structural loci of protein-protein interactions and examples of our GFP assay results. (a) Crystal structure (PDB id: 3W4U) depicting a D100Y mutation (on Hbb) at an interface residue and a F104L mutation in the interface domain for the Hbb-Hbz interaction. (b) Crystal structure (PDB id: 1G3N) depicting a V31L mutation (on Cdkn2c) away from the Cdkn2c-Cdk6 interaction interface. (c) GFP assays that determine the stability of wild-type Rrm2b and the R41P and L317V mutations on Rrm2b that are at an interface residue and away from the interface for the Rrm2b-Rrm2b interaction; GFP assays that determine the stability of wild-type Hprt1 and the C206Y mutation on Hprt1 that is away from the interaction interface of Hprt-Hprt1. Empty vector was used as a negative control.

For the 51 selected interactions, we chose 27 disease-associated mutations of residues at the interface (“interface residue”), 100 mutations in the rest of the interface domain (“interface domain”) and 77 mutations away from the interface (“away from the interface”; **Fig. 3a,b**). These interfaces were determined using solvent accessible surface area calculations as previously described [17], [18] on 7,340 co-crystal structures (**Materials and Methods**). To set up our Clone-seq pipeline, we first started with 39 mutations from these 204 and picked 4 colonies for each mutation. As a reference, we also pooled together all the wild-type alleles in our human ORFeome library to be sequenced together with the 4 pools of the mutagenesis colonies. In total, there were 40.1 million Illumina HiSeq 1×100 bp reads for our Clone-seq samples (**Text S1**) for an average of >2,500× coverage on all desired mutation sites. Therefore, our Clone-seq pipeline has the capacity to generate >3,000 mutations in one full lane of a HiSeq run with 1×100 bp reads, drastically improving the throughput and decreasing overall sequencing costs by at least 10-fold (**Text S1**).

Fig. 2a presents a schematic of the criteria we use to determine which clones contain the desired mutation and can be used for subsequent steps. For example, in pool 1, all reads (ignoring sequencing errors) confirm that genes I and II each contain the desired mutation – T116A and G298T, respectively. For gene III, we want to generate two separate clones with two separate mutations – III_A41T_ and III_C194T_. Since half the reads contain T41 (instead of A41) and the other half contain T194 (instead of C194), and we normalize DNA concentrations across all samples, we can infer that both mutant clones were generated successfully. In contrast, for gene IV, we see that while half the reads contain A511 (instead of G511), all the reads are wild-type at C74. Thus, we infer that while the IV_G511A_ clone is successfully generated, the IV_C74T_ clone is not. For gene V, although both mutant clones are successfully generated, half the reads contain an additional mutation, C436G. Since it is impossible to know which of the two clones for V contains this unwanted mutation, neither clone is usable. Similarly, we can determine mutant clones I_T116A_, III_A41T_, III_C194T_, IV_C74T_, IV_G511A_, V_T53G_, and V_G272A_ as usable clones in pool *n*. Based on these criteria, we developed the *S* score calculation and used it to determine successful mutagenesis attempts (**Materials and Methods**). Out of 156 colonies for 39 mutations, 125 of them contain the desired mutations (*S*>0.8), an overall 80% PCR-mutagenesis success rate. In fact, we were able to pick correct clones for all 39 mutant alleles using only the first two pools in Clone-seq. All 78 clones from the first two pools, from which the correct ones were selected for use in subsequent steps, were also Sanger sequenced for verification. 55 Clone-seq positive results with *S*>0.8 were all confirmed and there is a clear separation in the *S* scores between the successful and failed mutagenesis attempts (**Fig. 2b**).

One major advantage of our Clone-seq pipeline is that it allows us to carefully examine whether other unwanted mutations have been inadvertently introduced during PCR-mutagenesis in comparison with the corresponding wild-type alleles, since we obtain reads spanning the entire gene. We found that there are on average 4–5 unwanted mutations introduced in each pool of 39 colonies. This corresponds to a 0.013% PCR error rate (**Materials and Methods**), in agreement with previous studies [19]. The detection of unwanted mutations, especially those distant from the mutation of interest, is achieved in traditional site-directed mutagenesis pipelines by Sanger sequencing through the gene of interest. This is costly and labor-intensive, especially because multiple sequencing runs are needed for one long gene. However, since Clone-seq yields reads spanning the entire gene, we were able to determine which of the generated clones definitely do not have unwanted mutations in the full length of their sequences as illustrated in Fig. 2a (**Materials and Methods**), and we pick only these clones for subsequent assays.

To further test our Clone-seq pipeline, we applied it to generate clones for 113 SNPs on 66 genes from the recently published Exome Sequencing Project dataset [3]. Using the same approach as described above, we sequenced 4 colonies each for the 113 alleles of interest using one third of a 1×100 bp MiSeq run. We obtained 4.7 million reads for these 113 alleles. With a threshold of *S*>0.8, we were able to determine that 370 out of the 452 colonies (82%) contain the desired mutation, in perfect agreement with the PCR-mutagenesis success rate obtained earlier. We were able to choose colonies that contain only the desired mutation for all 113 alleles. Because the whole MiSeq run produced 17.7 million reads and we only used 4.7 million for generating the 113 mutant clones, the capacity of our Clone-seq pipeline using one full lane of a 1×100 bp HiSeq run is estimated to be >3,000, exactly the same as our previous assessment (**Text S1)**.

Finally, we generated the remaining 165 disease mutations (of the 204) and 717 other coding variants from the Exome Sequencing Project and the Catalog of Somatic Mutations in Cancer [20] using a full 1×100 bp HiSeq run, including 40 mutations on a single gene – *MLH1*. Using 111.2 million reads for these 882 alleles, we found that 2,958 of the 3,528 colonies (84%) contain the desired mutation, again in excellent agreement with our previously obtained PCR-mutagenesis success rate. There was at least one colony with only the desired mutation for all 882 alleles, including all 40 MLH1 mutations (**Table S1**). Therefore, our Clone-seq pipeline can generate a large number of mutations (>40) even for a single gene. In fact, to generate even more mutations for one gene, we can implement a two-round barcoding approach: generate groups of 40 mutations and barcode them differently for one HiSeq run (**Figure S3**). Ten such groups will enable us to generate ∼400 mutations for a single gene (**Text S1**). Since the average coverage of these 882 alleles is >300×, the capacity of our Clone-seq pipeline using one full lane of a 1×100 bp HiSeq run is estimated to be >3,000, again in agreement with our previous two estimates (**Text S1**).

Overall, our pipeline has been significantly optimized to make it very efficient. We established a web tool (http://www.yulab.org/Supp/MutPrimer) to design mutagenesis primers both individually and in batch. MutPrimer can design ∼1,000 primers for ∼500 mutations in one batch in less than one second. All of the 2,068 primers for the 1,034 mutations in this study were generated by MutPrimer. All mutagenesis PCRs are performed in batch using automatic 96-well procedures. Since single colony picking after bacterial transformation of mutagenesis PCR product is a rate-limiting step, we rigorously optimized this step and found that adding 10 µL mutagenesis PCR products to 100 µL competent cells and plating 50 µL transformed cells give the best transformation yield and well-separated single colonies. Furthermore, rather than individually streaking transformed cells onto agar plates one sample at a time, we were able to significantly increase throughput by spreading colonies using glass beads onto four sector agar plates which are partitioned into four non-contacting quadrants (**Materials and Methods**). In this manner, a 96-well plate of transformed bacteria can be plated out onto 24 four-sector agar plates in ∼15 minutes. Traditional site-directed mutagenesis pipelines require miniprepping each of the selected colonies and sequencing them separately by Sanger sequencing. To drastically improve the throughput of our Clone-seq pipeline, we pooled together the bacteria stock of a single colony for each mutagenesis attempt to perform one single maxiprep, which makes the library construction step much more efficient and amenable to high-throughput (**Text S1**). Furthermore, existing variant calling pipelines [21] cannot be applied to our Clone-seq results because the expected allelic ratios built into these pipelines are a function of the ploidy of the organism. However, in our Clone-seq pipeline there is no concept of ploidy. We pool together many mutations for one gene in the same pool (e.g., 40 mutations for *MLH1*) and different genes often have different numbers of mutations. Our *S* score calculation and unwanted mutation detection pipeline was designed according to our pooling strategy (**Materials and Methods**).

In total, we have used the novel Clone-seq pipeline successfully to generate 1,034 (39+113+882) mutant clones without any additional unwanted mutations, confirming the scalability, accuracy, and throughput of our Clone-seq pipeline.

### A high-throughput GFP assay to determine the impact of mutations on protein stability

**Figure 4:**
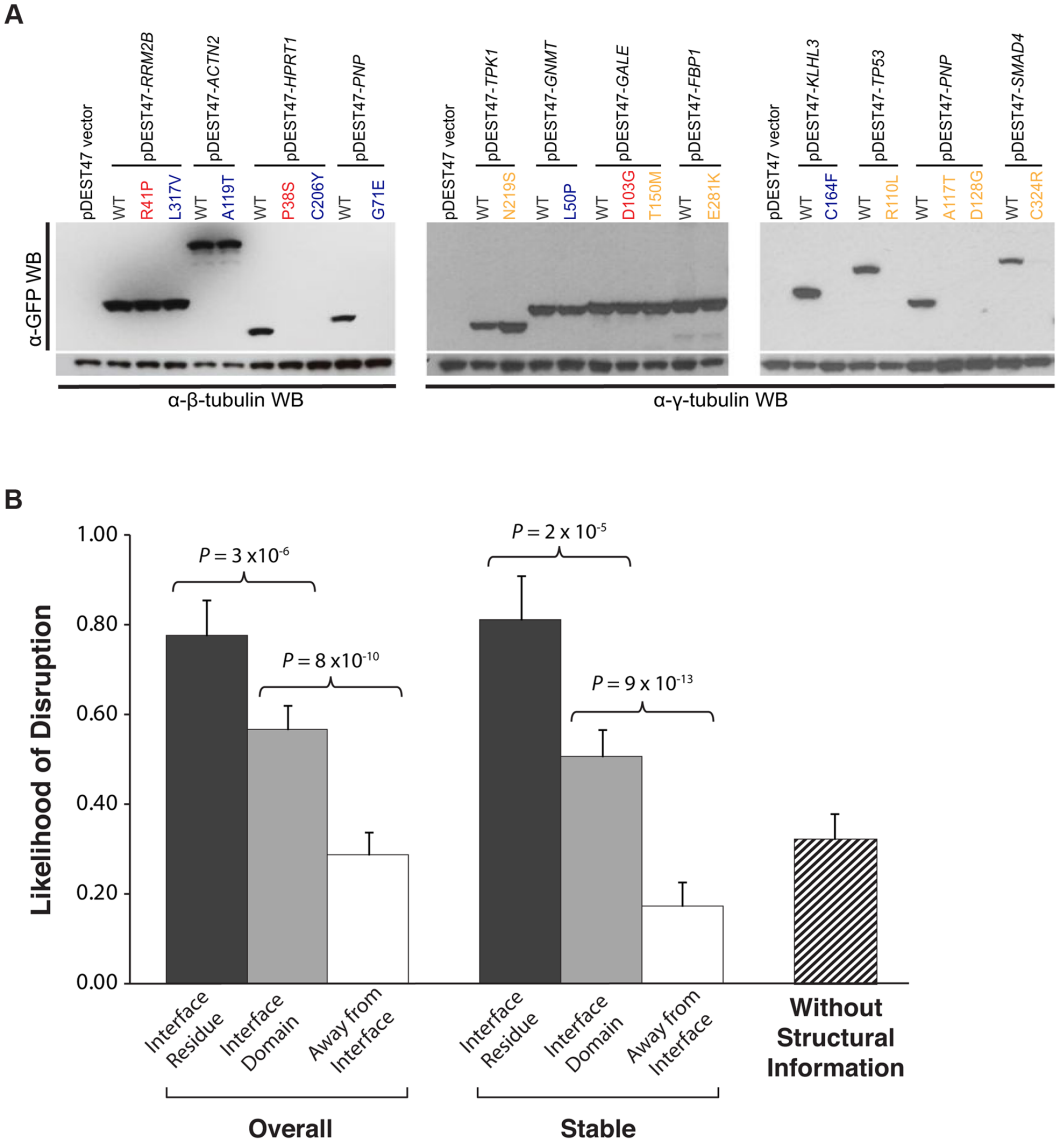
Effect of disease mutations on protein stability and protein-protein interactions. (a) Western blotting with anti-GFP antibody confirming the protein expression levels of wild-type Rrm2b, Actn2, Hprt1, Pnp, Tpk1, Gnmt, Gale, Fbp1, Klhl3, Tp53, Pnp, Smad4, and corresponding mutant alleles. β-tubulin and γ-tubulin were used as loading controls. Red denotes “interface residue” mutations, orange denotes “interface domain” mutations and blue denotes “away from the interface” mutations. (b) Likelihood of disruption of interactions by “interface residue”, “interface domain” and “away from the interface” mutations – overall and for stable mutants only; likelihood of a disease mutation disrupting a given interaction in the absence of structural information. Error bars indicate +SE. (*N* = 204 mutations).

For the 204 mutations on proteins with co-crystal structures, we first examined whether the mutant proteins can be stably expressed in human cells. To do this, we tagged every wild-type and mutant protein with GFP at the C-terminus using high-throughput Gateway cloning (**Fig. 1b**). The GFP constructs were transfected into HEK293T cells and fluorescence intensities were measured by a plate reader (**Fig. 3c**; **Materials and Methods**). All fluorescence intensity readings were also confirmed manually under a microscope. Compared with the corresponding wild-type proteins, the expression levels of 3 of the 27 “interface residue” mutants, 8 of the 99 “interface domain” mutants and 6 of the 77 “away from the interface” mutants are significantly diminished (**Fig. 3c**; **Materials and Methods**; **S2 Table**). To validate these findings, we also performed Western blotting for 8 random mutants that are stably expressed and 8 random mutants with significantly diminished expression levels (**Fig. 4a**). Western blotting results confirm our GFP intensity readings.

### A high-throughput Y2H assay to determine the impact of mutations on protein interactions

Next, we investigated whether these mutations could affect protein-protein interactions using Y2H (**Fig. 1c**; **Materials and Methods**). We found that 21 of the 27 (78%) “interface residue” mutations, 57 of the 100 (57%) “interface domain” mutations, and only 22 of the 77 (29%) “away from the interface” mutations disrupt the corresponding interactions, thereby demonstrating a clear difference (**Fig. 4b**; *P* = 3×10^−6^ between “interface residue” and “interface domain” and *P* = 8×10^−10^ between “interface domain” and “away from the interface”) in terms of ability to interfere with protein-protein interactions between mutations at different structural loci within the same protein. Furthermore, comparing with the GFP results, we found that all destabilizing mutations were shown to disrupt the corresponding interactions in our Y2H experiments. By considering only the mutations that do not affect protein expression based on the GFP experiments, we found the same difference: 13 out of 18 (72%) “interface residue” stable mutations, 42 out of 83 (51%) “interface domain” stable mutations, and only 9 out of 52 (17%) “away from the interface” stable mutations disrupt the corresponding interactions (**Fig. 4b**; *P* = 2×10^−5^ between “interface residue” and “interface domain” and *P* = 9×10^−13^ between “interface domain” and “away from the interface”; **Table S2**). Since these interfaces are obtained from actual co-crystal structures, our results suggest that accurate structural information can help determine the functional impact of mutations on protein-protein interactions. Wild-type proteins corresponding to 113 of the 153 stably expressed mutant proteins also interact with other proteins as determined by our Y2H experiments (114 interactions in total, termed “other interactions”); however, for these interactions, there are currently no co-crystal structures available in the PDB. Using these other interactions, we calculated the likelihood of a given mutation disrupting a specific interaction without any structural information to be 32% (**Fig. 4b**).

### Relationships between measured molecular phenotypes and corresponding disease phenotypes

**Figure 5:**
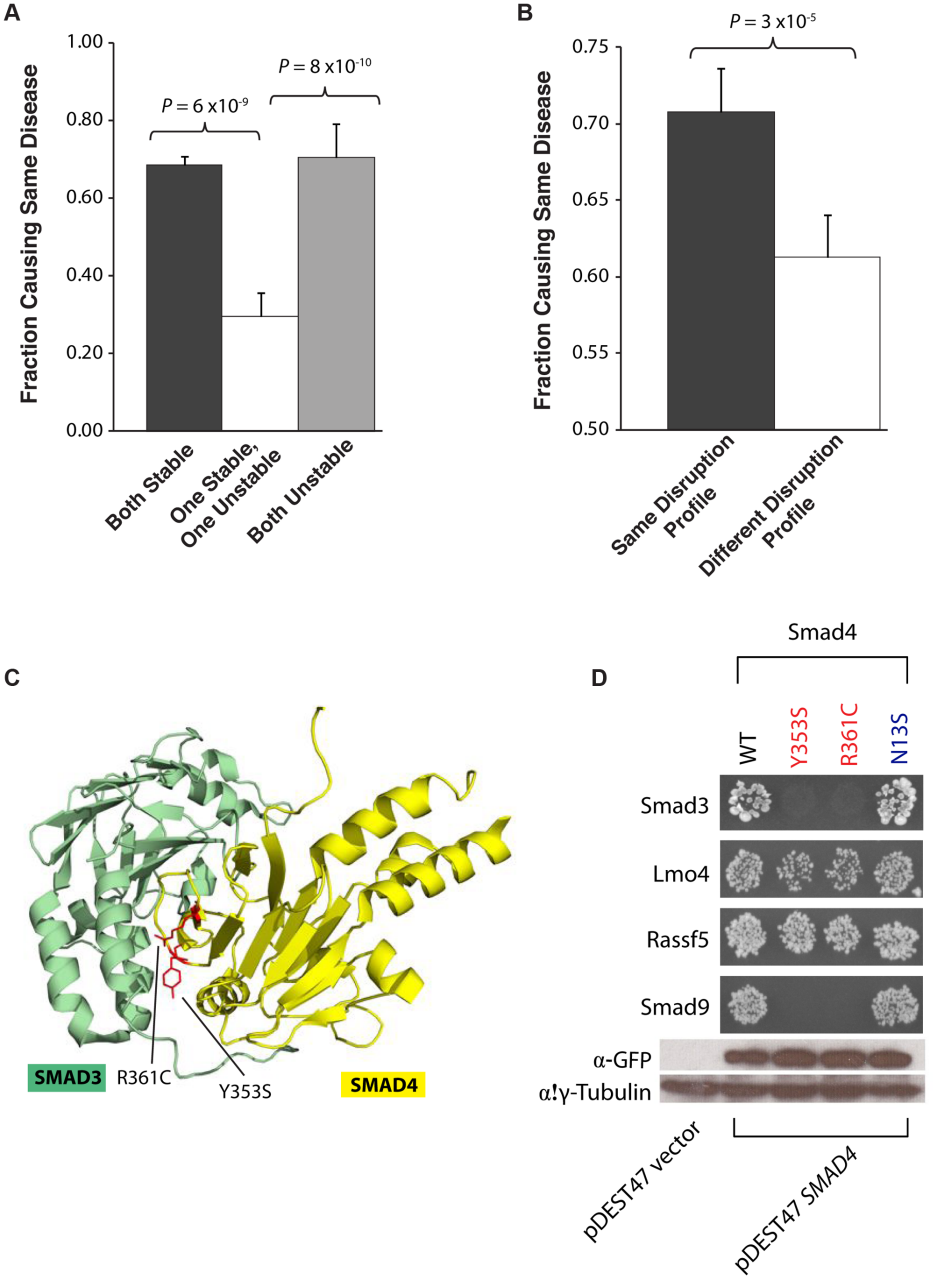
Relationships between molecular phenotypes and disease phenotypes. (a) Fraction of mutation pairs on the same gene that cause the same disease: for the same and different effects on protein stability. (b) Fraction of mutation pairs on the same gene that cause the same disease: for the same and different interaction disruption profiles. Error bars indicate +SE. (c) Crystal structure (PDB id: 1U7F) depicting the Y353S and R361C mutations (on Smad4) at interface residues for the Smad4-Smad3 interaction. (d) Y2H analysis of the effects of Smad Y353S, R361, and N13S mutations on its interactions with Smad3, Lmo4, Rassf5, and Smad9. Western blotting with anti-GFP antibody confirming the protein expression levels of wild-type Smad4 and its 3 mutant alleles – Y353S, R361C and N13S. γ-tubulin was used as a loading control.

We then analyzed whether the molecular phenotypes measured by our high-throughput GFP and Y2H assays are correlated with corresponding disease phenotypes. We first examined how mutation pairs on the same gene affect protein stability and its relationship to their corresponding diseases. We find that pairs of mutations that are either both stable or both unstable cause the same disease in 68% and 70% of cases, respectively. However, pairs comprising one stable and one unstable mutation cause the same disease in only 30% of cases (*P* = 6×10^−9^ and 8×10^−10^, respectively, **Fig. 5a**). For example, we find that the mutations R727C and L844F on the spindle checkpoint kinase Bub1b both cause the protein to become unstable and lose all its interactors. These mutations are both associated with the same disease, mosaic variegated aneuploidy, an autosomal recessive disorder that causes predominantly trisomies and monosomies of different chromosomes [22], [23]. Since our GFP assay shows that these two mutations cause loss of protein product, our results are consistent with Matusuura et al.'s finding that a more than 50% decrease in Bub1b activity leads to abnormal mitotic spindle checkpoint function and mosaic variegated aneuploidy [24].

We then examined whether mutation pairs on the same gene disrupt the same set or different sets of interactions (i.e., their interaction disruption profiles) and investigated whether their disruption profiles correlates with disease phenotypes. We found that mutation pairs with the exact same disruption profile are significantly more likely to cause the same disease than those with different profiles (70% and 61% respectively, *P* = 3×10^−5^, **Fig. 5b**). For example, we found that two mutations on Smad4, R361C and Y353S, disrupt its interactions with Smad3 and Smad9 while leaving the interactions with Lmo4 and Rassf5 unaltered (**Fig. 5c**). These two mutations both cause juvenile polyposis coli [25], [26], a disease is known to be caused by disruption of the core Smad/Bmp signaling pathways [27]. Our Y2H results clearly demonstrate that the R361C and Y353S mutations disrupt the Smad4-Smad3 and Smad4-Smad9 interactions (**Fig. 5c**) leading to disruption of core Smad signaling pathways. However, the mutation N13S on Smad4 does not disrupt any of these interactions (**Fig. 5c**) and is associated with a different disease, pulmonary arterial hypertension. Our results agree with Nasim et al.'s finding that the N13S mutation does not alter downstream Smad signaling [28]. Our findings provide support for the hypothesis that the N13S mutation either impacts pathways outside the core Smad signaling network or are pathogenic only when combined with other environmental and genetic factors [29].

Overall, these results show that mutation pairs with similar molecular phenotypes in terms of both protein stability and interactions are significantly more likely to cause the same disease than those with different molecular phenotypes. This confirms that the molecular phenotypes measured by our high-throughput GFP and Y2H assays are biologically relevant *in vivo*. Furthermore, by comparing the molecular phenotypes, in particular the protein interaction disruption profiles, of mutations/variants to those of known disease mutations, potential candidate mutations for a variety of diseases can be identified.

### A high-throughput mass spectrometry assay to determine the impact of mutations on protein interactions

**Figure 6:**
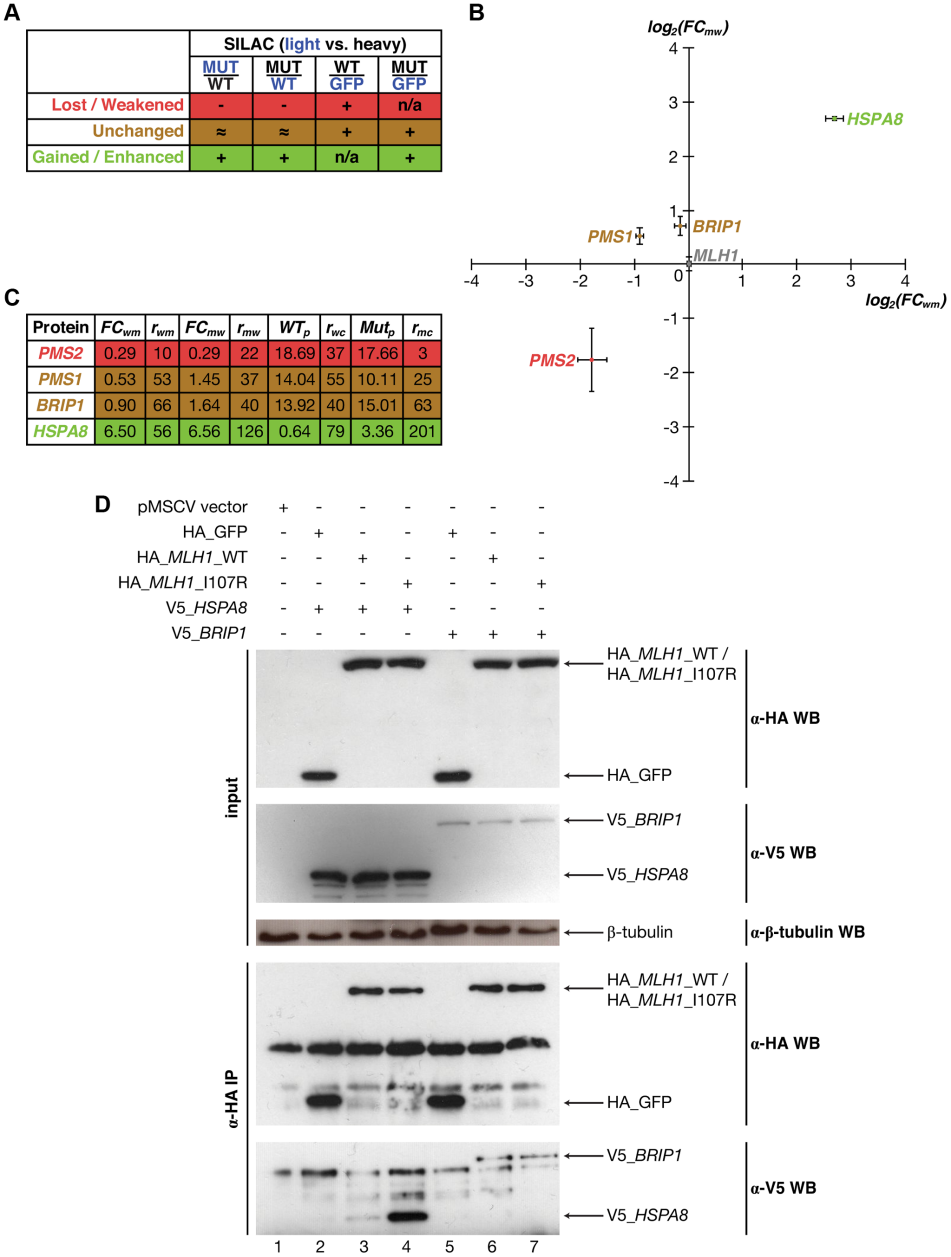
Identifying interactions of Mlh1 that are affected by the I107R mutation using SILAC-based mass spectrometry. (a) Schematic illustrating criteria used to identify interactions that are lost/weakened, unchanged, and gained/enhanced due to the I107R mutation on Mlh1. Blue denotes samples cultured in light media and black denotes samples cultured in heavy media. (b) Scatter plot illustrating fold change (*FC*; log scale) in the amount of protein pulled down by wild-type Mlh1 and mutant Mlh1 (I107R). Values are computed based on the wild-type (heavy) vs. mutant (light) (X-axis) and mutant (heavy) vs. wild-type (light) (Y-axis) experiments. Green denotes enhancement of interaction, red denotes weakening of interaction, and gold denotes no change. Mlh1 is shown in grey. (c) Fold changes and read counts (*r*) for interactors of Mlh1 that can be reliably identified as weakened, unchanged, and enhanced due to the I107R mutation. (d) Anti-HA immunoprecipitation followed by Western blotting with anti-V5 antibody confirming that the Mlh1-Brip1 interaction remains unchanged and that the Mlh1-Hspa8 interaction is dramatically enhanced due to the I107R mutation.

While we use only those interactions that are supported by co-crystal structures to estimate the fraction of interactions that are disrupted by mutations at different structural loci, the described procedures can also be applied to interactions with predicted interfaces and structural models [30], [31], [32], [33]. This is of particular importance because over 90% of known interactions do not currently have corresponding co-crystal structures [33], [34]. For example, Mlh1 is known to interact with Pms2, both of which are well-studied DNA mismatch repair genes frequently mutated in hereditary nonpolyposis colorectal cancer [35]. Although the structural basis of the Mlh1-Pms2 interaction still remains unknown, both our previous 3D reconstruction of the human interactome network [7], [32] and the newly-established Interactome3D [33] database suggest that the HATPase_c domain is part of the interface for Mlh1's interaction with Pms2. Previous work has shown that a point mutation (I107R) on the HATPase_c domain of Mlh1 is associated with colorectal cancer and disrupts the Mlh1-Pms2 interaction [7], [35], [36]. First, using Y2H, we were able to confirm the disruption (**Figure S4**). Next, we developed a high-throughput-amenable mass spectrometry pipeline using Stable Isotope Labeling by Amino acids in Cell culture (SILAC) [37], [38], which was designed to reveal both lost/weakened and gained/enhanced interactions of the target proteins (**Fig. 1d**) [39]. We added an HA-tag to the N-terminus of both wild-type and mutant Mlh1, as well as to GFP as a control, and performed four SILAC experiments: wild-type Mlh1 (heavy) vs. GFP control (light), mutant Mlh1 (heavy) vs. GFP control (light), wild-type (heavy) vs. mutant (light) Mlh1, and mutant (heavy) vs. wild-type (light) Mlh1 (**Fig. 6a**; **Materials and Methods**). Interactors of wild-type/mutant Mlh1 are defined as those that bind wild-type/mutant Mlh1 more than 2× stronger than GFP control (**Materials and Methods**). For a lost/weakened interaction, we required that the interaction be more than 2× stronger with wild-type Mlh1 than with mutant Mlh1 as confirmed both in wild-type (heavy) vs. mutant (light) and in mutant (heavy) vs. wild-type (light) experiments; we further required that the interaction be detected in the wild-type vs. control experiment (**Fig. 6a**; **Materials and Methods**). For a gained/enhanced interaction, we required that the interaction be more than 2× stronger with mutant Mlh1 than with wild-type Mlh1 as confirmed both in wild-type (heavy) vs. mutant (light) and in mutant (heavy) vs. wild-type (light) experiments; we further required that the interaction be detected in the mutant vs. control experiment (**Fig. 6a**; **Materials and Methods**). We were able to detect Pms2 as the only specifically weakened interactor caused by the mutation (**Figs. 6b,c**; *E* = −1.77; *P* = 3×10^−4^), in agreement with our Y2H results and previous studies [7], [36]. Additionally, we were able to detect Hspa8 as the only specifically enhanced interactor of the mutant protein (**Figs. 6b,c**; *E* = 2.71; *P* = 7×10^−8^). Two other known interactors of Mlh1, Pms1 (**Figs. 6b,c**; *E* = −0.32; *P* = 0.21) [40] and Brip1 (**Fig. 6b,c**; *E* = 0.18; *P* = 0.32) [41], were also detected, although their interactions with Mlh1 are not affected by this particular mutation (**Materials and Methods**).

Hspa8 was not previously known to interact with Mlh1 and the impact of the Mlh1 I107R mutation on its interactions with Pms1 and Brip1 has not been reported in the literature. To verify our SILAC results, we performed *in vivo* co-immunoprecipitation using HA-tagged wild-type and mutant Mlh1 and tagged Hspa8 and Brip1 with V5 (**Materials and Methods**). Our co-immunoprecipitation results confirm that Hspa8 only weakly interacts with wild-type Mlh1, but the interaction is dramatically enhanced by a single amino acid substitution (I107R) (**Fig. 6d**, lanes 3 and 4), whereas the interaction between Mlh1 and Brip1 is not affected by this mutation (**Fig. 6d**, lanes 6 and 7; **Materials and Methods**). Hspa8 is a constitutively expressed member of the heat shock protein 70 family [42]. It functions as a chaperone to facilitate protein folding [42] and also functions as an ATPase in the disassembly of clathrin-coated vesicles during membrane trafficking [43]. A recent study reported that Hspa8 is specifically recruited to reovirus viral factories, independent of its chaperone function [44]. Our Western blotting results demonstrate that the expression level of Mlh1 is not affected by the I107R mutation (**Figure S5**). Therefore, our SILAC results suggest that Hspa8 may play an important role in colorectal cancer and that its function could be independent of its role as a chaperone.

## Discussion

We have successfully developed the first massively parallel site-directed mutagenesis pipeline, Clone-seq, using next-generation sequencing. Our Clone-seq pipeline is entirely different from previously described random mutagenesis approaches [45], [46], [47], [48]. Clone-seq is used to generate a large number of specific mutant clones with desired mutations; each individual mutant clone has a separate stock and different clones can therefore be used separately for completely different downstream assays. In random mutagenesis, a pool of sequences containing different mutations for one gene is generated using error-prone PCR or error-prone DNA synthesis. Therefore, it is not possible to separate one mutant sequence from another and the whole pool can only be used for the same assay(s) together. Furthermore, it is not possible to control which or how many mutations are generated on each DNA sequence. In fact, to improve coverage, most random mutagenesis pipelines generate on average two or more mutations on each DNA sequence [45], which makes it impossible to distinguish the functional impact of each individual mutation on the same sequence. Site-directed mutagenesis and random mutagenesis are designed for different goals: if one wants to generate all possible mutations for a certain protein without the need to separate different clones, it would be more favorable to use random mutagenesis; whereas if one needs to have separate clones for each mutation, site-directed mutagenesis is required. As a result, the two approaches are complementary and not comparable.

While there are highly efficient methods for random mutagenesis [45], [46], [47], [48], current protocols for site-directed mutagenesis are low-throughput and become prohibitively expensive if a large number of clones needs to be generated. Clone-seq directly addresses the necessity for a high-throughput site-directed mutagenesis pipeline. It is a robust, cost-effective and efficient method that can be used to generate a total of ∼3,000 distinct mutant clones in one full lane of a 1×100 bp HiSeq run. Clone-seq is suitable both for generating mutations across many genes as well as a large number of mutations on a few genes. The former situation is applicable when one wants to generate many mutations/variants from large-scale studies (e.g., whole-genome or whole-exome sequencing) since they typically identify mutations/variants on a large number of genes [49], [50]. The latter situation usually arises in a study focused on a single pathway with a few genes of interest (e.g., an alanine-scanning mutagenesis to determine functional sites on a gene of interest [51]).

Integrating with Clone-seq, we also established a comprehensive comparative interactome-scanning pipeline, including high-throughput GFP, Y2H, and mass spectrometry assays, to systematically evaluate the impact of human disease mutations on protein stability and interactions. We examine each mutation individually, rather than looking at their combinatorial effects because these inherited germline disease mutations are extremely rare. Therefore, the probability of having even two of these in the same individual becomes infinitesimally small. Our results reveal that the overall likelihood of a given disease mutation disrupting a specific interaction is 32%. Accurate structural information of these interactions obtained from co-crystal structures greatly improves our understanding of the impact of disease mutations: 13 out of 18 (72%) “interface residue” stable mutations, 42 out of 83 (51%) “interface domain” stable mutations, and only 9 out of 52 (17%) “away from the interface” stable mutations disrupt the corresponding interactions, unveiling a clear dependence of the molecular phenotypes of disease mutations on their structural loci. These estimates are not affected by the false negative rate of our Y2H assay as we only use those interactions for which we can detect the wild-type interaction with strong Y2H phenotypes. Thus, any observed disruption is due to the mutation of interest and not an assay false negative. Furthermore, our Y2H pipeline has been shown to be of high quality and has an experimentally measured false positive rate of ∼5% or lower in different organisms [9], [12], [52], [53]. In addition, the interactions used to understand the relationship between molecular phenotypes and structural loci of disease mutations are all supported by co-crystal structures, therefore these interactions are not assay false positives. We also find that the molecular phenotypes detected by our GFP and Y2H assays correlate with known disease phenotypes, confirming the *in vivo* biological significance of our measurements.

Moreover, as shown by the Mlh1 example (**Fig. 6**), our comparative interactome-scanning pipeline can also be used with predicted structural models [30], [31], [32], [33]. The consequent experimental results will clearly be affected by the quality of these predictions, which is not part of our pipeline. In fact, our experimental interactome-scanning pipeline can be applied to evaluate or improve these predicted models by testing mutations at different loci of a protein of interest and examining how these mutations disrupt different interactions of this protein.

Our comparative interactome-scanning pipeline described and validated here can be applied to experimentally determine in a high-throughput fashion the impact on protein stability and protein-protein interactions for thousands of DNA coding variants and disease mutations, which can directly lead to hypotheses of concrete molecular mechanisms for follow-up studies. Furthermore, the elucidation of molecular phenotypes of disease mutations is also vital for selecting actionable drug targets and ultimately for making therapeutic decisions. Finally, the general scheme of our pipeline can be readily expanded to other interactome-mapping methods, particularly other protein-protein [10], protein-DNA [54], [55], protein-RNA [56], and protein-metabolite interaction assays [57], to comprehensively evaluate the functional relevance of all DNA variants, including those in non-coding regions.

## Materials and Methods

### Selecting interactions with mutations on and away from the interface

To calculate atomic-resolution interaction interfaces, we systematically examined a comprehensive list of 7,340 PDB co-crystal structures. To define the interface, we used a water molecule of diameter 1.4 Å as a probe and calculated the relative solvent accessible surface areas of the interacting pair as well as the individual proteins involved in the interaction. Residues whose relative accessibilities change by more than 1 Å^2^ are considered as potential interface residues, because amino acids at the interface reside on the surfaces of the corresponding proteins, but will tend to become buried in the co-crystal structure as the two proteins bind to each other [58]. So, for these residues, there should be a significant decrease in accessible surface area when we compare the bound and unbound states of the protein chains.

To identify interface domains, we required at least one of the following criteria to hold:

1. 3did [59] or iPfam [60] have identified the domain pair as interacting and each of the interface domains contains at least one interface residue based on our calculations.
2. The domain pair contains 5 or more interface residues for each protein according to our calculations.

We then identified the subset of these interactions that contain at least one disease mutation and are amenable to our version of Y2H [11], [12], [13], [14]. Subsequently, we performed a pairwise retest of all these interactions and selected the ones that yield strong Y2H phenotypes, because subsequent steps involve detecting a significant decrease in these phenotypes.

### Primer design for site-directed mutagenesis

Primers for site-directed mutagenesis were selected based on a customized version of the protocol accompanying the Stratagene QuikChange Site-Directed Mutagenesis Kit (200518). The following criteria are used:

1. The primer should be of length 30–50 bp and should contain the mutation of interest in the center or one base away.
2. The GC content of the primer should be ≥40% and the primer should start and end with a G or a C.
3. The T_m_ for the primer should be ≥78°C. T_m_ was calculated using the following expression:
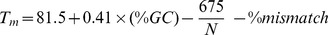
where *N* is the primer length in bases, *%GC* is the percentage of G or C nucleotides in the primer, and *%mismatch* is the percentage of mismatched bases in the primer. Values for *%GC* and *%mismatch* are whole numbers.

For cases where no primer satisfies all three criteria simultaneously, we relaxed criterion 2 to GC content ≥30%.

We established a supplementary web tool (http://www.yulab.org/Supp/MutPrimer) to design mutagenesis primers individually or in bulk.

### Construction of mutant alleles using high-throughput site-directed mutagenesis PCR

All wild-type clones were obtained from the human ORFeome v8.1 collection [61]. To generate mutant alleles, sequence-verified single-colony wild-type clones and their corresponding mutagenic primers were aliquoted into individual wells of 96-well PCR plates. Mutagenesis PCR was then performed as specified by the New England Biolabs (NEB) PCR protocol for Phusion polymerase (M0530L), noting that PCR was limited to 18 cycles. The samples were then digested by *Dpn*I (NEB R0176L) according to the manufacturer's manual. After digestion, samples were transformed into competent *E. coli*. Since single colony picking after bacterial transformation of mutagenesis PCR product is a rate-limiting step, we rigorously optimized this step. First, we tried different volumes of competent cells for transformation and found that single colony yields peak when ∼100 µL of competent cells are used. It is also necessary to use ∼10 µL of mutagenesis PCR product: any lower volume of PCR product results in significantly reduced colony yields, while higher volumes of PCR product do not increase yield. Finally, colony picking was done using four-sector agar plates (VWR 25384-308) that are partitioned into four non-contacting quadrants with glass beads poured onto each plate quadrant. Each bead-filled quadrant was inoculated with ∼50 µL of transformed bacteria. This was then spread by lightly shaking the four-sector agar plate. Our optimized transformation protocol results in a large number of well-separated single colonies that can be easily picked the next day. Upon recovery, single colonies from each quadrant were then picked and arrayed into 96-deepwell plates filled with 300 µL of antibiotic media. Four colonies per allele were picked for next-generation sequencing.

### DNA library preparation for Illumina sequencing

DNA library preparation was performed using NEBNext DNA Library Prep Master Mix Set for Illumina (NEB E6040S) according to the manufacturer's manual. Briefly, 5 µg of pooled plasmid DNA (∼100 µL, all samples were normalized to the same concentration) was sonicated to ∼200 bp fragments. The fragmented DNA was first mixed with NEBNext End Repair Enzyme for 30 mins at 20°C. Blunt-ended DNA was then incubated with Klenow Fragment for 30 mins at 37°C for dA-Tailing. Subsequently, NEBNext Adaptor was added to dA-Tailed DNA. Adaptor-ligated DNA (∼300 bp) was size-selected on a 2% agarose gel. Size-selected DNA was then mixed with one of the NEBNext Multiplex Oligos (NEB E7335S) and Universal PCR primers for PCR enrichment. At each step, DNA was purified using a QIAquick PCR purification kit (Qiagen 28104). Multiplexed DNA samples were combined and analyzed in one lane of a 1×100 bp run by Illumina HiSeq 2500.

### Identifying successful instances of site-directed mutagenesis based on next-generation sequencing

The mutant colonies were barcoded and pooled as shown in Fig. 1a. The multiplexed colonies were then run on an Illumina sequencer (2 HiSeq runs and 1 MiSeq run) to give 1×100 bp reads. These reads were then de-multiplexed and mapped to the genes of interest using the BWA “aln” algorithm [62]. For each allele, we identified all reads that mapped to the position of the mutation of interest (*R_all_*) and those that actually contained the desired mutation (*R_mut_*). We then calculated a normalized score (*S*) that quantifies the fraction of reads containing the desired mutation:
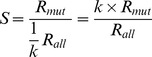
where *k* is the number of different mutations for the same gene.

For 39 mutations, we Sanger sequenced two mutant colonies per mutagenesis attempt to quantify the correlation between *S* and observation of the desired mutation. We found that all clones with *S*>0.44 are confirmed to be correct via Sanger sequencing with a clear separation between those that are correct and those that are not (**Fig. 2b**). However, to further ensure that the clones we picked were correct, we require *S*>0.8 for a colony to be scored as containing the desired mutation.

### Identifying unwanted mutations

One major advantage of our Clone-seq pipeline over traditional site-directed mutagenesis protocols using Sanger sequencing [15] is that we can now carefully examine whether there are other unwanted mutations inadvertently introduced during the PCR process, in comparison with the corresponding wild-type alleles. It is essential to use clones with no unwanted mutations for downstream experiments, as the presence of these will make it impossible to determine whether the observed disruption is due to the desired or other undesirable mutation(s).

We use samtools “mpileup” [63] to obtain read counts for different alleles at each nucleotide for all the clones. We calculate the background sequencing error rate by calculating the average fraction of non-reference alleles across all nucleotides where we did not attempt to introduce a mutation. Any site that has a significantly higher fraction of non-reference alleles (using a *P* value cutoff of 0.2 from a cumulative binomial test) is considered to have an unwanted mutation. A lenient *P* value cutoff (0.2 as opposed to the more traditionally used 0.05 or 0.01) implies more stringent filtering in this case because we want to eliminate type II errors i.e., we want to identify all unwanted mutations at the cost of discarding a few clones that actually do not have any unwanted mutations.

We identified an average of 4–5 unwanted point mutations per pool. The overall per-base point mutation rate of Phusion polymerase was calculated to be ∼10^−4^. NEB's advertised error rate for Phusion polymerase varies from 4.4–9.5×10^−7^ per PCR cycle. Since we perform 18 PCR cycles, the expected overall error rate is ∼10^−5^. Our calculated mutation is within an order of magnitude of this advertised error rate. It is slightly higher than the advertised rate as we use stringent filtering criteria as described above.

### GFP assay

All wild-type and mutant clones were moved into the pcDNA-DEST47 vector with a C-terminal GFP tag using automated Gateway LR reactions in a 96-well format. After bacterial transformation, minipreps were prepared on a Tecan Freedom Evo 200, and DNA concentrations were determined by OD 260/280 with a Tecan Infinite M1000 plate reader in 96-well format. A 100 ng aliquot of each expression clone plasmid was used for transfection into HEK293T cells in 96-well plates using Lipofectamine 2000 (Invitrogen 11668019) according to the manufacturer's instructions. At approximately 48 hrs post-transfection, cells were processed with Tecan M1000. Fluorescence intensities were measured at 395 nm for excitation and 507 nm for emission, according to Invitrogen's manual. As negative controls, the fluorescence intensities corresponding to cells transfected with the empty vector were measured. The normalized fluorescence intensity was calculated as:
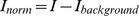
where *I* corresponds to the measured intensity and *I_background_* corresponds to the average intensity of the empty vector controls for each plate. All *I_norm_* values greater than *K* are considered to correspond to stable protein expression. *K* corresponds to the range (maximum – minimum) of background fluorescence intensities of the empty vector controls for each plate. For this study, all fluorescence intensity readings were also confirmed manually under a microscope. All transfection and GFP experiments were repeated 3 times.

### Y2H assay

Y2H was performed as previously described [7]. All wild-type/mutant clones were transferred by Gateway LR reactions into our Y2H pDEST-AD and pDEST-DB vectors. All DB-X and AD-Y plasmids were transformed individually into the Y2H strains *MAT*α Y8930 and *MAT* **a** Y8800, respectively. Each of the DB-X *MAT*α transformants (wild-type and mutants) were then mated against corresponding AD-Y *MAT* **a** transformants (wild-type and mutants) individually using automated 96-well procedures, including inoculation of AD-Y and DB-X yeast cultures, mating on YEPD media (incubated overnight at 30°C), and replica-plating onto selective Synthetic Complete media lacking leucine, tryptophan, and histidine, and supplemented with 1 mM of 3-amino-1,2,4-triazole (SC-Leu-Trp-His+3AT), SC-Leu-His+3AT plates containing 1 mg/l cycloheximide (SC-Leu-His+3AT+CHX), SC-Leu-Trp-Adenine (Ade) plates, and SC-Leu-Ade+CHX plates to test for CHX-sensitive expression of the *LYS2::GAL1-HIS3* and *GAL2-ADE2* reporter genes. The plates containing cycloheximide select for cells that do not have the AD plasmid due to plasmid shuffling. Growth on these control plates thus identifies spontaneous auto-activators [64]. The plates were incubated overnight at 30°C and “replica-cleaned” the following day. Plates were then incubated for another three days, after which positive colonies were scored as those that grow on SC-Leu-Trp-His+3AT and/or on SC-Leu-Trp-Ade, but not on SC-Leu-His+3AT+CHX or on SC-Leu-Ade+CHX. Disruption of an interaction by a mutation was defined as at least 50% reduction of growth consistently across both reporter genes, when compared to Y2H phenotypes of the corresponding wild-type allele as benchmarked by 2-fold serial dilution experiments. All Y2H experiments were repeated 3 times.

### Construction of plasmids

Wild-type *MLH1*, *HSPA8*, and *BRIP1* entry clones are from the human ORFeome v8.1 collection [61]. Using Gateway LR reactions, wild-type *MLH1*, mutant *MLH1* (I107R), and GFP were transferred into the pMSCV-N-FLAG-HA-PURO vector [65]; *HSPA8* and *BRIP1* were transferred into the pcDNA-DEST40 vector that contains a C-terminal V5 tag (Invitrogen 12274-015).

### Analysis of interacting proteins by SILAC and LC-MS/MS

HEK293T cells were grown in SILAC media comprising SILAC DMEM (Thermo Scientific) and 10% dialyzed FBS (JR Scientific) supplemented with either 0.1 mg/ml L-lysine and L-arginine (light media) or 0.1 mg/ml L-lysine 13C6, 15N2 and L-arginine 13C6, 15N4 (heavy media). Heavy- or light-media cultured HEK293T cells were transfected using Lipofectamine 2000 (Invitrogen) in three 10 cm plates. 48 hrs after transfection, cells were washed three times in cold PBS and then resuspended in 5 ml RIPA buffer [1% NP-40, 50 mM Tris-HCl pH 7.5, 150 mM NaCl, 5 mM EDTA, 1× EDTA-free Complete Protease Inhibitor tablet (Roche)]. Cells were lysed for 30 mins on ice before centrifuging at 13,000 rpm for 10 mins. Cell lysates were incubated with 60 µL EZview Red Anti-HA Affinity Gel (Sigma-Aldrich) for 3 hrs. After 3 washes with RIPA buffer, bound proteins were eluted with 3 resin volumes elution buffer (100 mM Tris-HCl pH 8.0, 1% SDS). Eluted proteins from light and heavy media were mixed together, reduced with 5 mM DTT, alkylated with 15 mM of iodoacetamide, and then precipitated with 3 volumes PPT solution (50% acetone, 49.9% ethanol, 0.1% acetic acid). Proteins from pull-down experiments were solubilized with 50 µL Urea/Tris solution (8 M Urea, 50 mM Tris-HCl pH 8.0) and 150 µL NaCl/Tris (50 mM Tris-HCl pH 8.0, 150 mM NaCl) followed by the addition of 1 µg Trypsin Gold (Promega). Protein digestion was performed overnight at 37°C after which trifluoroacetic acid and formic acid were added to a final concentration of 0.2%. Peptides were de-salted with Sep-Pak C18 columns (Waters Corporation), dried in a speed-vac, and reconstituted in 85 µL of a solution containing 80% acetonitrile and 1% formic acid. Samples were fractionated by Hydrophilic Interaction LIquid Chromatography (HILIC) using a TSK gel Amide-80 column (Tosoh Bioscience). HILIC fractions were dried in a speed-vac, reconstituted in 0.1% trifluoroacetic acid, and analyzed by LC-MS/MS using a 125 µM ID capillary column packed in-house with 3 µm C18 particles (Michrom Bioresources) and a Q-Exactive mass spectrometer (Thermo Fisher Scientific) coupled with a Nano LC-Ultra system (Eksigent). Xcalibur 2.2 software (Thermo Fischer Scientific) was used for the data acquisition and Q-Exactive was operated in the data-dependent mode. Survey scans were acquired in the Orbitrap mass analyzer over the range of 380 to 2000 m/z with a mass resolution of 70.000 (at m/z 200). Up to the top 10 most abundant ions with a charge state higher than 1 and less than 5 were selected within an isolation window of 2.0 m/z. Selected ions were fragmented by Higher-energy Collisional Dissociation (HCD) and the tandem mass spectra were acquired in the Orbitrap mass analyzer with a mass resolution of 17.500 (at m/z 200). The fragmentation spectra were searched by using the SEQUEST software on a SORCERER system (Sage-N Research) and a human database downloaded from the International Protein Index (version 3.80). In all database searches, trypsin was designated as the protease, allowing for one non-tryptic end and two missed-cleavages. The following parameters were used in the database search: a mass accuracy of 15 ppm for the precursor ions, differential modification of 8.0142 Daltons for lysine and 10.00827 Daltons for arginine. Results were filtered based on probability score to achieve a 1% false positive rate. The Xpress software, part of the Trans-Proteomic Pipeline (Seattle Proteome Center), was used to process the raw data and quantify the light/heavy peptide isotope ratios. Results were also manually inspected.

### Identifying loss and gain of interactors for Mlh1

We performed four SILAC experiments using both wild-type and mutant Mlh1, as well as GFP as a control: wild-type (heavy) vs. control (light) [WT_Control]; mutant (heavy) vs. control (light) [Mutant_Control]; wild-type (heavy) vs. mutant (light) [WT_Mutant]; and mutant (heavy) vs. wild-type (light) [Mutant_WT].

We use the following variables and define four ratios for all subsequent calculations. In the WT_Control experiment, the relative abundance of protein *p* pulled down by wild-type Mlh1 to protein *p* pulled down by GFP (*WT_p_*) is quantified by the inverse of the geometric mean of *r_wc_* reads with Xpress values *X_i_*. In the Mutant_Control experiment, the relative abundance of protein *p* pulled down by mutant Mlh1 (I107R) to protein *p* pulled down by GFP (*Mut_p_*) is quantified by the inverse of the geometric mean of *r_mc_* reads with Xpress values *Y_i_*. In the WT_Mutant experiment, the relative abundance of protein *p* pulled down with mutant Mlh1 (I107R) to protein *p* pulled down by wild-type Mlh1 is quantified by the geometric mean of *r_wm_* reads with Xpress values *P_i_*. The amount of mutant Mlh1 (I107R) to wild-type Mlh1 is quantified by the geometric mean of *t_wm_* reads with Xpress values *C_j_*. In the Mutant_WT experiment, the relative abundance of protein *p* pulled down with mutant Mlh1 (I107R) to protein *p* pulled down by wild-type Mlh1 is quantified by the inverse of the geometric mean of *r_mw_* reads with Xpress values *Q_j_*. The amount of mutant Mlh1 (I107R) to wild-type Mlh1 is quantified by the inverse of the geometric mean of *t_mw_* reads with Xpress values *D_i_*.
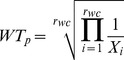


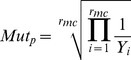


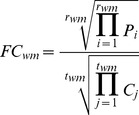


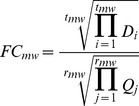
where both *FC_wm_* and *FC_mw_* denote the fold change in protein abundance as the normalized ratio of the amount of protein pulled down with mutant Mlh1 to that with wild-type Mlh1.

To identify interactors that are lost/weakened due to the I107R mutation, we required the following criteria to hold simultaneously:

1. The protein has to be identified as an interactor of wild-type Mlh1: *WT_p_*>2, *r_wc_*≥5.
2. The protein has to be identified as a lost interactor based on both Mutant_WT: *FC_mw_*<0.5, *r_mw_*≥5, and WT_Mutant: *FC_wm_*<0.5, *r_wm_*≥5.

The first criterion ensures that the protein identified is a true interactor of wild-type Mlh1. The second criterion ensures that the loss of interaction is significant and reliably observed across both WT_Mutant and Mutant_WT experiments.

Similarly, to identify interactors that are gained/enhanced due to the I107R mutation, we required the following criteria to hold simultaneously:

1. The protein has to be identified as an interactor of mutant Mlh1 (I107R): *Mut_p_*>2, *r_mc_*≥5.
2. The protein has to be identified as a gained interactor based on both Mutant_WT: *FC_mw_*>2, *r_mw_*≥5, and WT_Mutant: *FC_wm_*>2, *r_wm_*≥5.

The first criterion ensures that the protein identified is a true interactor of the I107R mutant of Mlh1. The second criterion ensures that the gain of interaction is significant and reliably observed across both WT_Mutant and Mutant_WT experiments.

We also identify interactors of Mlh1 that are unaffected by the I107R mutation using the following criteria:

1. The protein has to be identified as an interactor of both wild-type Mlh1: *WT_p_*>2, *r_wc_*≥5, and mutant Mlh1 (I107R): *Mut_p_*>2, *r_mc_*≥5.
2. The protein has to be identified as an unchanged interactor based on both Mutant_WT: 0.5<*FC_mw_*<2, *r_mw_*≥5, and WT_Mutant: 0.5<*FC_wm_*<2, *r_wm_*≥5.

Integrating both WT_Mutant and Mutant_WT experiments, we calculated a weighted average of the individual fold changes:
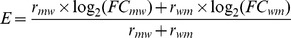

*P* values are calculated using a two-sided Kolmogorov-Smirnov test (with bootstrapping).

### Cell culture, co-immunoprecipitation, and Western blotting

HEK293T cells were maintained in complete DMEM medium supplemented with 10% FBS. Cells were transfected with Lipofectamine 2000 (Invitrogen) at a 6∶1 (µL/µg) ratio with DNA in 6-well plates and were harvested 24 hrs after transfection. Cells were gently washed three times in PBS and then resuspended using 200 µL 1% NP-40 lysis buffer [1% Nonidet P-40, 50 mM Tris-HCl pH 7.5, 150 mM NaCl, 1× EDTA-free Complete Protease Inhibitor tablet (Roche)] and kept on ice for 20 mins. Extracts were cleared by centrifugation for 10 mins at 13,000 rpm at 4°C. 15 µL EZview Red Anti-HA Affinity Gel (Sigma-Aldrich) and 100 µL protein lysate were used for each co-immunoprecipitation reaction. The samples were rotated gently at 4°C for 2 hrs. HA beads were then washed three times with protein lysis buffer, treated with 6× protein sample buffer, and subjected to SDS-PAGE. Proteins were then transferred from the gel onto PVDF (Amersham) membranes. Anti-HA (Sigma H9658), anti-V5 (Invitrogen 46-0705), anti-β-tubulin (Promega G7121), and anti-GFP (Santa Cruz sc-9996) antibodies were used at 1∶3,000 dilutions for immunoblotting analysis.

## Supporting Information

Figure S1 Number of high-quality binary interactions detected by various assays.
(TIFF)

Figure S2 Schematic of steps used to select mutations and interactions for our comparative interactome-scanning pipeline.
(TIFF)

Figure S3 Schematic illustrating our two round barcoding approach to generate groups of 40 mutations and barcode them differently for one HiSeq run.
(TIFF)

Figure S4 Y2H assay showing that the *MLH1*-*PMS2* interaction is weakened by the I107R mutation on *MLH1*.
(TIFF)

Figure S5 Western blot demonstrating the protein expression level of *MLH1* is not affected by the I107R mutation.
(TIFF)

Table S1 Summary of the 4 colonies for each of the 40 mutations generated on *MLH1*. “1” indicates that for the mutation of interest, mutagenesis PCR succeeded for that colony and “0” indicates that for the mutation of interest, the mutagenesis PCR failed for that colony. None of the colonies contained any unwanted mutations on *MLH1*.
(XLSX)

Table S2 Summary of GFP and Y2H assay results for all the mutations tested in our interactome-scanning pipeline. For the GFP assay: “1” indicates a stable mutation, “0” indicates an unstable mutation, and “–” indicates inconclusive results due to weak signal for the wild-type protein. For the Y2H assay: “1” indicates no disruption and “0” indicates disruption of the corresponding interaction.
(XLSX)

Text S1 Supplementary text.
(PDF)

## References

[1] Stenson PD, Mort M, Ball EV, Howells K, Phillips AD, et al (2009) The Human Gene Mutation Database: 2008 update. Genome Med 1: 13. 19348700 10.1186/gm13 PMC2651586

[2] Consortium TGP (2012) An integrated map of genetic variation from 1,092 human genomes. Nature 491: 56–65. 23128226 10.1038/nature11632 PMC3498066

[3] Fu W, O'Connor TD, Jun G, Kang HM, Abecasis G, et al (2013) Analysis of 6,515 exomes reveals the recent origin of most human protein-coding variants. Nature 493: 216–220. 23201682 10.1038/nature11690 PMC3676746

[4] Hindorff LA, Sethupathy P, Junkins HA, Ramos EM, Mehta JP, et al (2009) Potential etiologic and functional implications of genome-wide association loci for human diseases and traits. Proc Natl Acad Sci U S A 106: 9362–9367. 19474294 10.1073/pnas.0903103106 PMC2687147

[5] Vidal M, Cusick ME, Barabasi AL (2011) Interactome networks and human disease. Cell 144: 986–998. 21414488 10.1016/j.cell.2011.02.016 PMC3102045

[6] Zhong Q, Simonis N, Li QR, Charloteaux B, Heuze F, et al (2009) Edgetic perturbation models of human inherited disorders. Mol Syst Biol 5: 321. 19888216 10.1038/msb.2009.80 PMC2795474

[7] Wang X, Wei X, Thijssen B, Das J, Lipkin SM, et al (2012) Three-dimensional reconstruction of protein networks provides insight into human genetic disease. Nat Biotechnol 30: 159–164. 22252508 10.1038/nbt.2106 PMC3708476

[8] Berman HM, Westbrook J, Feng Z, Gilliland G, Bhat TN, et al (2000) The Protein Data Bank. Nucleic Acids Res 28: 235–242. 10592235 10.1093/nar/28.1.235 PMC102472

[9] Yu H, Braun P, Yildirim MA, Lemmens I, Venkatesan K, et al (2008) High-quality binary protein interaction map of the yeast interactome network. Science 322: 104–110. 18719252 10.1126/science.1158684 PMC2746753

[10] Braun P, Tasan M, Dreze M, Barrios-Rodiles M, Lemmens I, et al (2009) An experimentally derived confidence score for binary protein-protein interactions. Nat Methods 6: 91–97. 19060903 10.1038/nmeth.1281 PMC2976677

[11] Rual JF, Venkatesan K, Hao T, Hirozane-Kishikawa T, Dricot A, et al (2005) Towards a proteome-scale map of the human protein-protein interaction network. Nature 437: 1173–1178. 16189514 10.1038/nature04209

[12] Venkatesan K, Rual JF, Vazquez A, Stelzl U, Lemmens I, et al (2009) An empirical framework for binary interactome mapping. Nat Methods 6: 83–90. 19060904 10.1038/nmeth.1280 PMC2872561

[13] Yu H, Tardivo L, Tam S, Weiner E, Gebreab F, et al (2011) Next-generation sequencing to generate interactome datasets. Nat Methods 8: 478–480. 21516116 10.1038/nmeth.1597 PMC3188388

[14] HI2012 (2012) http://interactomedfciharvardedu/indexphp?page=login&lg=/H_sapiens/indexphp?page=newrelease.

[15] Suzuki Y, Kagawa N, Fujino T, Sumiya T, Andoh T, et al (2005) A novel high-throughput (HTP) cloning strategy for site-directed designed chimeragenesis and mutation using the Gateway cloning system. Nucleic Acids Res 33: e109. 16009811 10.1093/nar/gni103 PMC1174934

[16] Salehi-Ashtiani K, Yang X, Derti A, Tian W, Hao T, et al (2008) Isoform discovery by targeted cloning, ‘deep-well’ pooling and parallel sequencing. Nat Methods 5: 597–600. 18552854 10.1038/nmeth.1224 PMC2743938

[17] Das J, Lee HR, Sagar A, Fragoza R, Liang J, et al (2014) Elucidating common structural features of human pathogenic variations using large-scale atomic-resolution protein networks. Hum Mutat 35: 585–593. 24599843 10.1002/humu.22534 PMC4876038

[18] Khurana E, Fu Y, Colonna V, Mu XJ, Kang HM, et al (2013) Integrative annotation of variants from 1092 humans: application to cancer genomics. Science 342: 1235587. 24092746 10.1126/science.1235587 PMC3947637

[19] Vandenbroucke I, Van Marck H, Verhasselt P, Thys K, Mostmans W, et al (2011) Minor variant detection in amplicons using 454 massive parallel pyrosequencing: experiences and considerations for successful applications. Biotechniques 51: 167–177. 21906038 10.2144/000113733

[20] Forbes SA, Bindal N, Bamford S, Cole C, Kok CY, et al (2011) COSMIC: mining complete cancer genomes in the Catalogue of Somatic Mutations in Cancer. Nucleic Acids Res 39: D945–950. 20952405 10.1093/nar/gkq929 PMC3013785

[21] McKenna A, Hanna M, Banks E, Sivachenko A, Cibulskis K, et al (2010) The Genome Analysis Toolkit: a MapReduce framework for analyzing next-generation DNA sequencing data. Genome Res 20: 1297–1303. 20644199 10.1101/gr.107524.110 PMC2928508

[22] Hanks S, Coleman K, Reid S, Plaja A, Firth H, et al (2004) Constitutional aneuploidy and cancer predisposition caused by biallelic mutations in BUB1B. Nat Genet 36: 1159–1161. 15475955 10.1038/ng1449

[23] Suijkerbuijk SJ, van Osch MH, Bos FL, Hanks S, Rahman N, et al (2010) Molecular causes for BUBR1 dysfunction in the human cancer predisposition syndrome mosaic variegated aneuploidy. Cancer Res 70: 4891–4900. 20516114 10.1158/0008-5472.CAN-09-4319 PMC2887387

[24] Matsuura S, Matsumoto Y, Morishima K, Izumi H, Matsumoto H, et al (2006) Monoallelic BUB1B mutations and defective mitotic-spindle checkpoint in seven families with premature chromatid separation (PCS) syndrome. Am J Med Genet A 140: 358–367. 16411201 10.1002/ajmg.a.31069

[25] Roth S, Sistonen P, Salovaara R, Hemminki A, Loukola A, et al (1999) SMAD genes in juvenile polyposis. Genes Chromosomes Cancer 26: 54–61. 10441006 10.1002/(sici)1098-2264(199909)26:1<54::aid-gcc8>3.0.co;2-d

[26] Houlston R, Bevan S, Williams A, Young J, Dunlop M, et al (1998) Mutations in DPC4 (SMAD4) cause juvenile polyposis syndrome, but only account for a minority of cases. Hum Mol Genet 7: 1907–1912. 9811934 10.1093/hmg/7.12.1907

[27] Massague J (2008) TGFbeta in Cancer. Cell 134: 215–230. 18662538 10.1016/j.cell.2008.07.001 PMC3512574

[28] Nasim MT, Ogo T, Ahmed M, Randall R, Chowdhury HM, et al (2011) Molecular genetic characterization of SMAD signaling molecules in pulmonary arterial hypertension. Hum Mutat 32: 1385–1389. 21898662 10.1002/humu.21605

[29] Machado RD (2012) The molecular genetics and cellular mechanisms underlying pulmonary arterial hypertension. Scientifica (Cairo) 2012: 106576. 24278664 10.6064/2012/106576 PMC3820608

[30] Tuncbag N, Gursoy A, Nussinov R, Keskin O (2011) Predicting protein-protein interactions on a proteome scale by matching evolutionary and structural similarities at interfaces using PRISM. Nat Protoc 6: 1341–1354. 21886100 10.1038/nprot.2011.367 PMC7384353

[31] Zhang QC, Petrey D, Deng L, Qiang L, Shi Y, et al (2012) Structure-based prediction of protein-protein interactions on a genome-wide scale. Nature 490: 556–560. 23023127 10.1038/nature11503 PMC3482288

[32] Meyer MJ, Das J, Wang X, Yu H (2013) INstruct: a database of high-quality 3D structurally resolved protein interactome networks. Bioinformatics 29: 1577–1579. 23599502 10.1093/bioinformatics/btt181 PMC3673217

[33] Mosca R, Ceol A, Aloy P (2013) Interactome3D: adding structural details to protein networks. Nat Methods 10: 47–53. 23399932 10.1038/nmeth.2289

[34] Das J, Fragoza R, Lee HR, Cordero NA, Guo Y, et al (2014) Exploring mechanisms of human disease through structurally resolved protein interactome networks. Mol Biosyst 10: 9–17. 24096645 10.1039/c3mb70225a PMC4061614

[35] Peltomaki P, Vasen HF (1997) Mutations predisposing to hereditary nonpolyposis colorectal cancer: database and results of a collaborative study. The International Collaborative Group on Hereditary Nonpolyposis Colorectal Cancer. Gastroenterology 113: 1146–1158. 9322509 10.1053/gast.1997.v113.pm9322509

[36] Kondo E, Suzuki H, Horii A, Fukushige S (2003) A yeast two-hybrid assay provides a simple way to evaluate the vast majority of hMLH1 germ-line mutations. Cancer Res 63: 3302–3308. 12810663

[37] Ong SE, Blagoev B, Kratchmarova I, Kristensen DB, Steen H, et al (2002) Stable isotope labeling by amino acids in cell culture, SILAC, as a simple and accurate approach to expression proteomics. Mol Cell Proteomics 1: 376–386. 12118079 10.1074/mcp.m200025-mcp200

[38] Ong SE, Mann M (2006) A practical recipe for stable isotope labeling by amino acids in cell culture (SILAC). Nat Protoc 1: 2650–2660. 17406521 10.1038/nprot.2006.427

[39] Ohouo PY, Bastos de Oliveira FM, Almeida BS, Smolka MB (2010) DNA damage signaling recruits the Rtt107-Slx4 scaffolds via Dpb11 to mediate replication stress response. Mol Cell 39: 300–306. 20670896 10.1016/j.molcel.2010.06.019

[40] Leung WK, Kim JJ, Wu L, Sepulveda JL, Sepulveda AR (2000) Identification of a second MutL DNA mismatch repair complex (hPMS1 and hMLH1) in human epithelial cells. J Biol Chem 275: 15728–15732. 10748105 10.1074/jbc.M908768199

[41] Peng M, Litman R, Xie J, Sharma S, Brosh RM Jr, et al (2007) The FANCJ/MutLalpha interaction is required for correction of the cross-link response in FA-J cells. EMBO J 26: 3238–3249. 17581638 10.1038/sj.emboj.7601754 PMC1914102

[42] Goldfarb SB, Kashlan OB, Watkins JN, Suaud L, Yan W, et al (2006) Differential effects of Hsc70 and Hsp70 on the intracellular trafficking and functional expression of epithelial sodium channels. Proc Natl Acad Sci U S A 103: 5817–5822. 16585520 10.1073/pnas.0507903103 PMC1458656

[43] DeLuca-Flaherty C, McKay DB, Parham P, Hill BL (1990) Uncoating protein (hsc70) binds a conformationally labile domain of clathrin light chain LCa to stimulate ATP hydrolysis. Cell 62: 875–887. 1975516 10.1016/0092-8674(90)90263-e

[44] Kaufer S, Coffey CM, Parker JS (2012) The cellular chaperone hsc70 is specifically recruited to reovirus viral factories independently of its chaperone function. J Virol 86: 1079–1089. 22090113 10.1128/JVI.02662-10 PMC3255859

[45] Fowler DM, Araya CL, Fleishman SJ, Kellogg EH, Stephany JJ, et al (2010) High-resolution mapping of protein sequence-function relationships. Nat Methods 7: 741–746. 20711194 10.1038/nmeth.1492 PMC2938879

[46] Starita LM, Pruneda JN, Lo RS, Fowler DM, Kim HJ, et al (2013) Activity-enhancing mutations in an E3 ubiquitin ligase identified by high-throughput mutagenesis. Proc Natl Acad Sci U S A 110: E1263–1272. 23509263 10.1073/pnas.1303309110 PMC3619334

[47] Araya CL, Fowler DM, Chen W, Muniez I, Kelly JW, et al (2012) A fundamental protein property, thermodynamic stability, revealed solely from large-scale measurements of protein function. Proc Natl Acad Sci U S A 109: 16858–16863. 23035249 10.1073/pnas.1209751109 PMC3479514

[48] Pitt JN, Ferre-D'Amare AR (2010) Rapid construction of empirical RNA fitness landscapes. Science 330: 376–379. 20947767 10.1126/science.1192001 PMC3392653

[49] Stransky N, Egloff AM, Tward AD, Kostic AD, Cibulskis K, et al (2011) The mutational landscape of head and neck squamous cell carcinoma. Science 333: 1157–1160. 21798893 10.1126/science.1208130 PMC3415217

[50] Atlas TCG (2012) Comprehensive molecular characterization of human colon and rectal cancer. Nature 487: 330–337. 22810696 10.1038/nature11252 PMC3401966

[51] Cunningham BC, Wells JA (1989) High-resolution epitope mapping of hGH-receptor interactions by alanine-scanning mutagenesis. Science 244: 1081–1085. 2471267 10.1126/science.2471267

[52] Consortium AIM (2011) Evidence for network evolution in an Arabidopsis interactome map. Science 333: 601–607. 21798944 10.1126/science.1203877 PMC3170756

[53] Das J, Vo TV, Wei X, Mellor JC, Tong V, et al (2013) Cross-species protein interactome mapping reveals species-specific wiring of stress response pathways. Sci Signal 6: ra38. 23695164 10.1126/scisignal.2003350 PMC3777727

[54] Reece-Hoyes JS, Barutcu AR, McCord RP, Jeong JS, Jiang L, et al (2011) Yeast one-hybrid assays for gene-centered human gene regulatory network mapping. Nat Methods 8: 1050–1052. 22037702 10.1038/nmeth.1764 PMC3263363

[55] Berger MF, Philippakis AA, Qureshi AM, He FS, Estep PW 3rd, et al (2006) Compact, universal DNA microarrays to comprehensively determine transcription-factor binding site specificities. Nat Biotechnol 24: 1429–1435. 16998473 10.1038/nbt1246 PMC4419707

[56] Yakhnin AV, Yakhnin H, Babitzke P (2012) Gel mobility shift assays to detect protein-RNA interactions. Methods Mol Biol 905: 201–211. 22736005 10.1007/978-1-61779-949-5_12 PMC4687016

[57] Bandyopadhyay A, Saxena K, Kasturia N, Dalal V, Bhatt N, et al (2012) Chemical chaperones assist intracellular folding to buffer mutational variations. Nat Chem Biol 8: 238–245. 22246401 10.1038/nchembio.768 PMC3527004

[58] Franzosa EA, Xia Y (2011) Structural principles within the human-virus protein-protein interaction network. Proc Natl Acad Sci U S A 108: 10538–10543. 21680884 10.1073/pnas.1101440108 PMC3127880

[59] Stein A, Ceol A, Aloy P (2011) 3did: identification and classification of domain-based interactions of known three-dimensional structure. Nucleic Acids Res 39: D718–723. 20965963 10.1093/nar/gkq962 PMC3013799

[60] Finn RD, Marshall M, Bateman A (2005) iPfam: visualization of protein-protein interactions in PDB at domain and amino acid resolutions. Bioinformatics 21: 410–412. 15353450 10.1093/bioinformatics/bti011

[61] Yang X, Boehm JS, Salehi-Ashtiani K, Hao T, Shen Y, et al (2011) A public genome-scale lentiviral expression library of human ORFs. Nat Methods 8: 659–661. 21706014 10.1038/nmeth.1638 PMC3234135

[62] Li H, Durbin R (2009) Fast and accurate short read alignment with Burrows-Wheeler transform. Bioinformatics 25: 1754–1760. 19451168 10.1093/bioinformatics/btp324 PMC2705234

[63] Li H, Handsaker B, Wysoker A, Fennell T, Ruan J, et al (2009) The Sequence Alignment/Map format and SAMtools. Bioinformatics 25: 2078–2079. 19505943 10.1093/bioinformatics/btp352 PMC2723002

[64] Walhout AJ, Vidal M (2001) High-throughput yeast two-hybrid assays for large-scale protein interaction mapping. Methods 24: 297–306. 11403578 10.1006/meth.2001.1190

[65] Behrends C, Sowa ME, Gygi SP, Harper JW (2010) Network organization of the human autophagy system. Nature 466: 68–76. 20562859 10.1038/nature09204 PMC2901998

