# Supplementary figures and images for: A Massively Parallel Pipeline to Clone DNA Variants and Examine Molecular Phenotypes of Human Disease Mutations
Source: PLoS Genet. 2014 Dec 11;10(12):e1004819. doi: 10.1371/journal.pgen.1004819 (PMC4263371; doi:10.1371/journal.pgen.1004819)

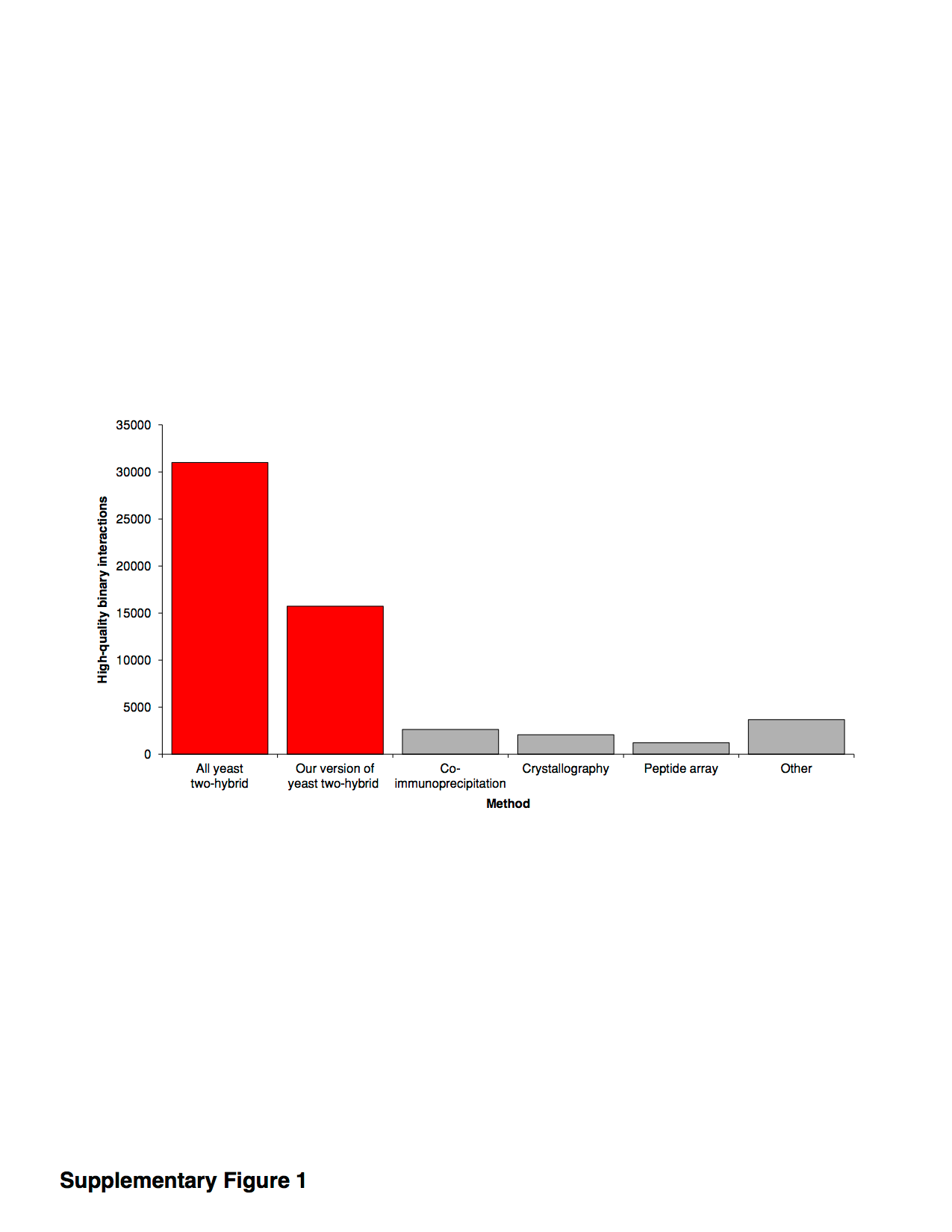

Supplement: Figure S1 — Number of high-quality binary interactions detected by various assays. (TIFF) [file pgen.1004819.s001.tiff]

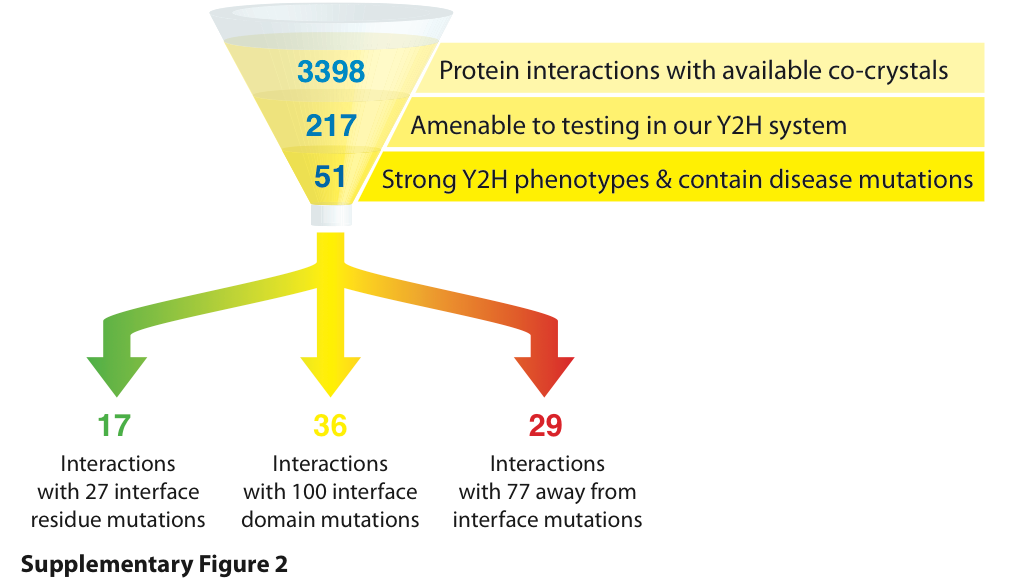

Supplement: Figure S2 — Schematic of steps used to select mutations and interactions for our comparative interactome-scanning pipeline. (TIFF) [file pgen.1004819.s002.tiff]

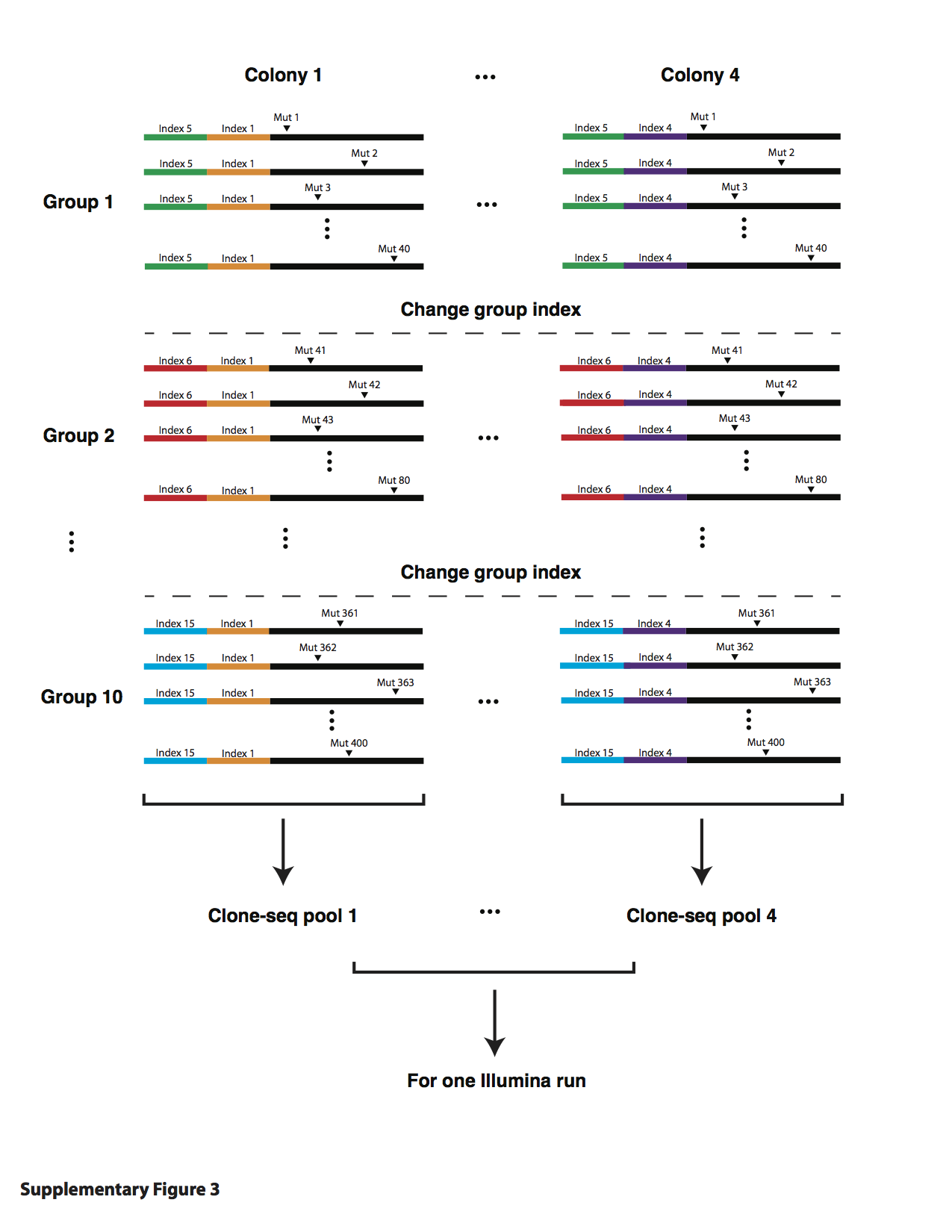

Supplement: Figure S3 — Schematic illustrating our two round barcoding approach to generate groups of 40 mutations and barcode them differently for one HiSeq run. (TIFF) [file pgen.1004819.s003.tiff]

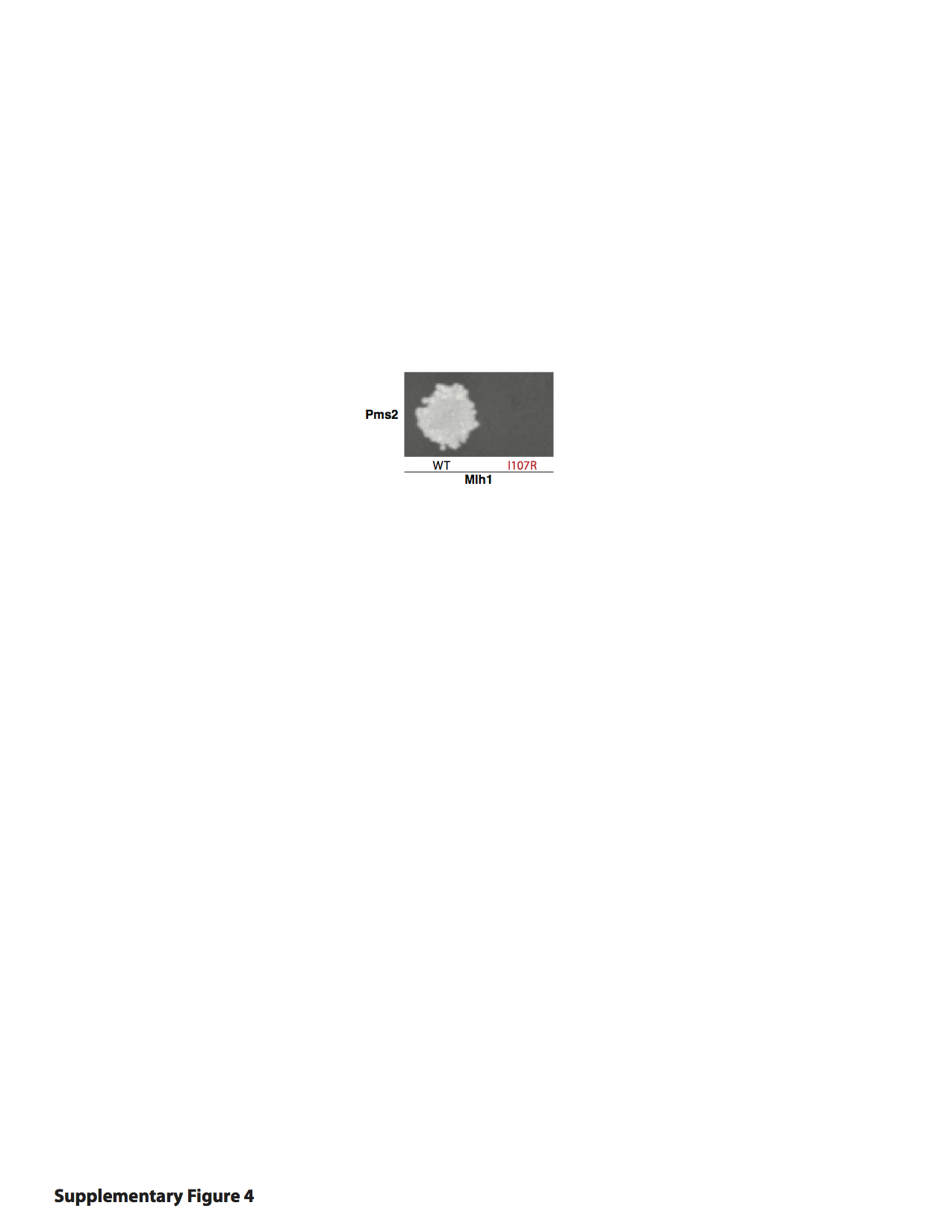

Supplement: Figure S4 — Y2H assay showing that the MLH1-PMS2 interaction is weakened by the I107R mutation on MLH1. (TIFF) [file pgen.1004819.s004.tiff]

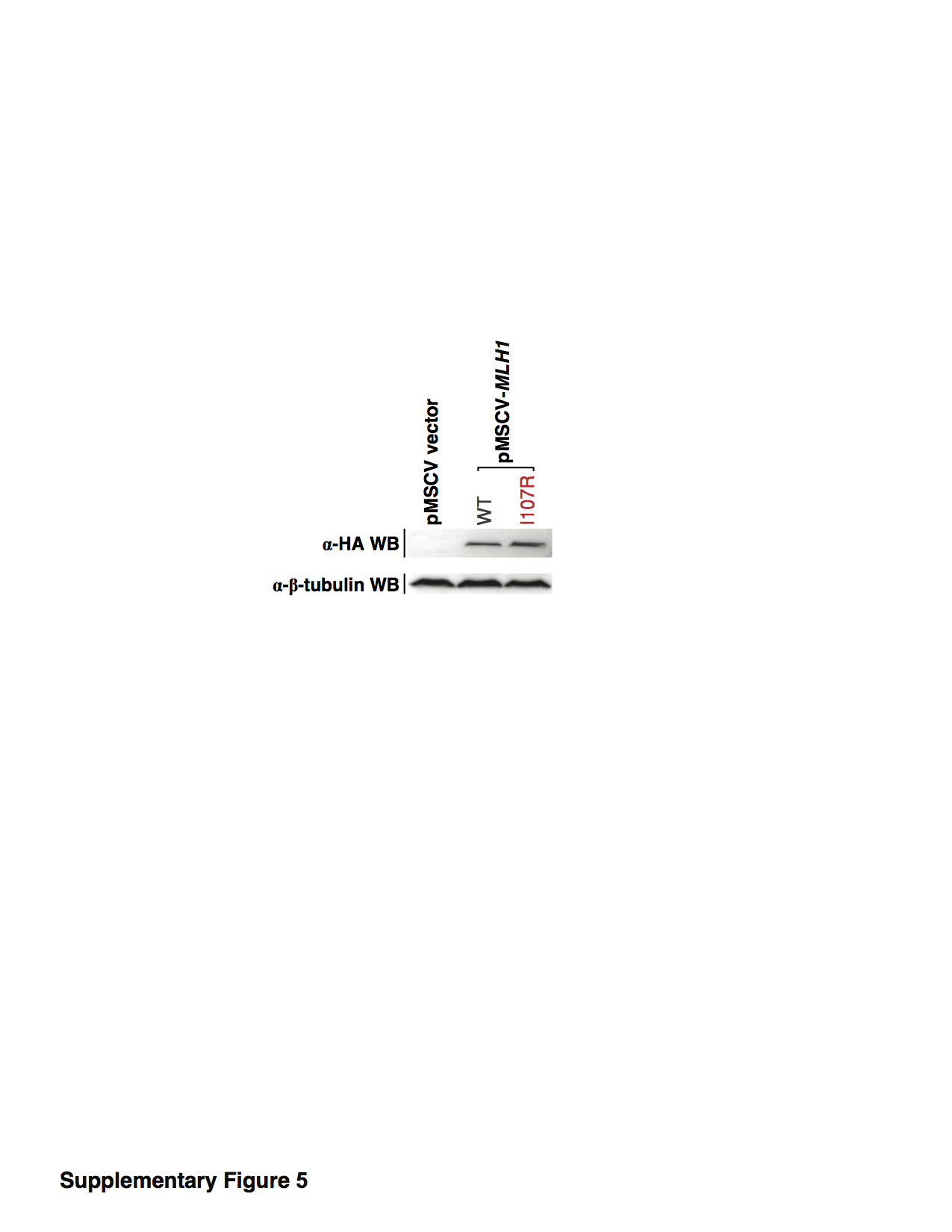

Supplement: Figure S5 — Western blot demonstrating the protein expression level of MLH1 is not affected by the I107R mutation. (TIFF) [file pgen.1004819.s005.tiff]
