# Supplementary material for: A Massively Parallel Pipeline to Clone DNA Variants and Examine Molecular Phenotypes of Human Disease Mutations
Source: PLoS Genet. 2014 Dec 11;10(12):e1004819. doi: 10.1371/journal.pgen.1004819 (PMC4263371; doi:10.1371/journal.pgen.1004819)
Supplement: Text S1 — Supplementary text. (PDF) [file pgen.1004819.s008.pdf]

Supplementary information for:

A massively parallel pipeline to clone DNA variants and examine molecular phenotypes of human disease mutations

Xiaomu Wei[1,2*], Jishnu Das[2,3*], Robert Fragoza[2,4*], Jin Liang[2*], Francisco M. Bastos de Oliveira[2,4], Hao Ran Lee[2,3], Xiujuan Wang[2,3], Matthew Mort[5], Peter D. Stenson[5], David N. Cooper[5], Steven M. Lipkin[1], Marcus B. Smolka[2,4], Haiyuan Yu[2,3¶]

[1]Department of Medicine, Weill Cornell College of Medicine, New York, NY 10021, USA; [2]Weill Institute for Cell and Molecular Biology, [3]Department of Biological Statistics and Computational Biology, [4]Department of Molecular Biology and Genetics, Cornell University, Ithaca, NY 14853, USA; [5]Institute of Medical Genetics, Cardiff University, Heath Park, Cardiff CF14 4XN, UK.

*These authors contributed equally to this work. [¶]To whom correspondence should be addressed. Email: haiyuan.yu@cornell.edu

Probability of obtaining the desired clone

If the probability of successfully obtaining the desired clone with a single colony is p, assuming independence between the colonies, the chance of not obtaining even one desired clone after picking n colonies is $(1 - p)^n$. Thus, the probability of obtaining at least one correct clone for one mutagenesis attempt is:

$$P(n) = 1 - (1 - p)^n$$

Based on our HiSeq results, we can estimate p using the average fraction of desired clones obtained. Since we sequenced four colonies for each of the 39 desired clones, we have a total of 156 samples. Out of the 156 samples, 125 contain the desired mutations. Thus, p = 125/156 = 0.8. Substituting appropriate values, we calculate the probability of obtaining at least one correct clone for one mutagenesis attempt after picking 4 colonies, $P(4) = 0.998$.

In our pipeline, even colonies for generating different mutations of the same gene can be put into the same pool, which can be easily distinguished computationally when processing the sequencing results. Confusion only arises upon pooling colonies for generating the same mutation with identical surrounding sequences in the same gene or between different genes. In this situation, we can only identify the correct clones if all of these mutations in the same pool are correct. However, out of 50,491 missense disease mutations in HGMD and 395,780 coding SNPs in dbSNP, only 340 (~0.08%) will cause such confusion in Clone-seq.

The probability of obtaining the desired clones for k instances of the same mutation with identical surrounding sequences in the same gene or between different genes is given by:

$$P(n,k) = 1 - (1 - p^k)^n$$

Thus, even if we were to have 3 undistinguishable mutations with identical surrounding sequences (i.e., $k = 3$), after picking 4 colonies for each mutagenesis attempt, we would still have a 94% chance to have at least one pool out of the four where all three mutations are correct, rendering the whole Clone-seq pipeline successful.

Scalability of Clone-seq

The primary determinant of the scalability of our Clone-seq pipeline is the read coverage for alleles that we generate using our high-throughput mutagenesis PCR protocol. The average coverage of reads for each of the 39 alleles in our Clone-seq results is > 2,500×. For our Clone-seq results, we only used ~40 million reads out of a total of ~125 million reads in a single lane of a 1×100 bp HiSeq run. So, if we use all 125 million reads for the 4 colonies, we can sequence 39×(125/40) alleles with > 2,500× coverage. However, to determine S to a least count of 1%, we only need 100× coverage. Since the separation between a successful mutagenesis attempt with the lowest S and an unsuccessful mutagenesis attempt with the highest S is 0.28, 100× coverage makes this separation > 25 times our least count. We further increase this separation to > 60 times our least count by requiring $S > 0.8$ for a mutagenesis attempt to be considered successful. 100× coverage is also sufficient for a conservative variant calling pipeline to identify additional mutations with high confidence [2,3]. Thus, we can generate 39×(125/40)×(2,500/100) = 3,047 mutant alleles with a single lane of a 1×100 bp HiSeq run using the Clone-seq pipeline.

Costs of Sanger sequencing vs. Clone-seq

Traditional Sanger sequencing	
Unique mutations	3,047
Colonies per mutation	4
Total number of samples	3,047×4 = 12,188
Re-sequencing needed[1]	5%
Number of 96-well plates needed	137
Cost per plate	$300
Minimum cost[2]	43×$300 = **$12,900**
Total cost	137×$300 = **$41,100**

Clone-seq	
NEBNext Multiplex Oligos (E7335S)	$19.80
NEBNext DNA Library Prep Master (E6040S)	$105
Illumina HiSeq, single-end, 100 bp sequencing lane	$1,175
Total cost	**$1,299.80**

All costs are based on internal Cornell pricing.
[1] Sanger sequencing has an average failure rate of 5%.
[2] The minimum cost is the least amount of money spent in Sanger sequencing the expected number of samples needed to obtain one correct clone for each mutation of interest. Since the PCR-mutagenesis success rate (p) is 0.8, the expected number of samples that need to be sequenced is given by:

$$E(\#\, Mut) = P(1) + n \times \left(\sum_{n=2}^{4} P(n) - P(n+1) \right)$$

where $P(n)$ is the probability of obtaining at least one correct clone after Sanger sequencing n colonies, which is calculated as $1 - (1 - p)^n$. However, the calculated minimum cost is only a lower bound estimation and the real cost is likely to be much higher. One main reason is that for mutations in the middle of long genes, internal sequencing primers are required.

Generating many mutations on the same gene using Clone-seq

To test the limit of Clone-seq, we attempted to generate 40 mutations on *MLH1*, together with 842 other mutations in one HiSeq run. With 4 colonies picked for each mutation, we were able to get at least one successful mutant clone with no additional unwanted mutations for all 40 mutations (Table S1). To generate even more mutations for the same gene, a two-round barcoding approach can be used. We can generate 10 groups of 40 mutations and barcode them a second time as shown in Fig. S3. Thus, all clones will have barcodes of the form $x.y$ where x varies between 5-14 and y varies between 1-4. The first part of the barcode (x) denotes the group, while the second part of the barcode (y) indicates the colony.

A single maxiprep to optimize library construction

Traditional site-directed mutagenesis pipelines require miniprepping each of the selected colonies and sequencing them separately by Sanger sequencing. To generate the batch of 882 clones using Clone-seq, we miniprepped all bacteria stocks individually before pooling the plasmids and barcoding for sequencing. For these 882 alleles, we found that 2,958 of the 3,528 colonies (84%) contain the desired mutation. This is in excellent agreement with the mutagenesis-PCR success rates for 2 other batches of clones (125/156 [80%] colonies containing the desired mutation and 370/452 colonies [82%] containing the desired mutation) that were generated using Clone-seq.

To drastically improve the throughput of our Clone-seq pipeline, we then pooled together the bacteria stock of a single colony for each mutagenesis attempt (882 in total) to perform one single maxiprep and found that the accuracy of clone generation stays the same – 665 of the 882 (75%) colonies contain the desired mutation. Since the accuracy of clone generation remains the same even when performing a single maxiprep, this strategy can be employed to make the library construction step much more efficient and amenable to high-throughput.
